# Therapeutic monoclonal antibody targeting of neuronal pentraxin receptor to control metastasis in gastric cancer

**DOI:** 10.1186/s12943-020-01251-0

**Published:** 2020-08-26

**Authors:** Mitsuro Kanda, Dai Shimizu, Koichi Sawaki, Shunsuke Nakamura, Shinichi Umeda, Takashi Miwa, Haruyoshi Tanaka, Chie Tanaka, Masamichi Hayashi, Yohei Iguchi, Suguru Yamada, Masahisa Katsuno, Yasuhiro Kodera

**Affiliations:** 1 Department of Gastroenterological Surgery (Surgery II), Nagoya University Graduate School of Medicine, 65 Tsurumai-cho, Showa-ku, Nagoya, 466-8550 Japan; 2 Department of Neurology, Nagoya University Graduate School of Medicine, Nagoya, Japan

**Keywords:** Gastric cancer, Neuronal pentraxin receptor, Antibody, Knockout mouse

## Abstract

**Background:**

Controlling metastasis is essential for improving the prognosis of patients with gastric cancer (GC). Here, we aimed to identify a molecule required for GC metastasis and to investigate its potential utility as a target for the development of therapeutic antibodies (Abs).

**Methods:**

Transcriptome and bioinformatics analyses of human GC cell lines identified the neuronal pentraxin receptor (*NPTXR*) as a candidate molecule. *NPTXR* function was probed by modulating its expression in GC cells and assessing the effects on intracellular signaling and malignant behaviors in vitro and in mouse xenograft models. We also generated anti-NPTXR Abs and *Nptxr*^*−/−*^ mice, and assessed the clinical significance of *NPTXR* expression in GC specimens.

**Results:**

*NPTXR* mRNA expression in clinical specimens was associated with disease progression and was significantly higher in tissues from GC patients with distant metastasis compared with those without. *NPTXR* regulated expression of genes involved in metastatic behaviors as well as activation of the PI3K–AKT–mTOR, FAK–JNK, and YAP signaling pathways. *NPTXR* silencing promoted caspase-mediated apoptosis and attenuated GC cell proliferation, cell cycle progression, migration, invasion, adhesion, stem cell-like properties, and resistance to 5-fluorouracil in vitro, and also inhibited the tumorigenicity of GC cells in vivo. Anti-*NPTXR* Abs inhibited GC peritoneal metastasis in mice. *Nptxr*^*−/−*^ mice showed no abnormalities in reproduction, development, metabolism, or motor function.

**Conclusions:**

*NPTXR* plays an essential role in controlling the malignant behavior of GC cells in vitro and in vivo. *NPTXR*-targeting Abs may thus have utility as novel diagnostic tools and/or treatment modalities for GC.

## Introduction

Although the incidence of gastric cancer (GC) has decreased over the past few decades, it is still one of the three most common malignancies worldwide and remains a global health burden [1]. GC can be treated when diagnosed sufficiently early, and patients undergoing resection can expect an excellent prognosis. However, patients diagnosed with advanced cancer face a dire prognosis, mainly because of the propensity for GC to metastasize [2].

The only therapeutic options currently available for patients with unresectable or metastatic GC are combinations of cytotoxic anti-cancer agents and targeted therapies such as monoclonal antibodies (mAbs) [3]. Trastuzumab (anti-Her2), ramucirumab (anti-vascular endothelial growth factor receptor 2), nivolumab (anti-PD-1), and pembrolizumab (anti-PD-L1) are mAbs that target growth factor receptors or immune checkpoints and have demonstrated efficacy for GC in large-scale clinical trials [4–6]. However, the efficacy and prognosis of these treatments are unpredictable owing to the clinical heterogeneity and molecular complexity of GC [7, 8]. Moreover, some mAbs are not well tolerated, leaving the patient with few treatment options [9]. There is thus an urgent need to identify candidate therapeutic targets for the development of agents that can control cancer metastasis through novel mechanisms.

To this end, we performed transcriptome and bioinformatics analysis of GC tissues from patients with or without metastasis to identify novel candidate targets involved in metastasis. We identified neuronal pentraxin receptor (*NPTXR*) as being specifically overexpressed in GC tissues with metastatic potential. *NPTXR* is a type II transmembrane protein that functions as a trans-synaptic organizer and anchors neuronal pentraxin complexes to plasma membranes [10, 11]. However, little is known about its possible roles in cancer [12]. We investigated the expression and function of *NPTXR* by in vitro and in vivo analysis of human GC cell lines, tumor xenograft mouse models, and *Nptxr*-deficient (*Nptxr*^−/−^) mice. We also developed polyclonal Abs (pAbs) and mAbs against NPTXR and evaluated their potential utility as diagnostic tools and/or therapeutic agents for GC.

## Materials and methods

More details were provided in Additional file 1.

### Cell lines and clinical samples

The GC cell lines GCIY, IM95, MKN1, MKN7, MKN45, MKN74, NUGC2, NUGC3, NUGC4, OCUM-1, and SC-6-JCK were obtained from the Japanese Collection of Research Bioresources Cell Bank (JCRB, Osaka, Japan). AGS, KATOIII, and N87 GC cell lines and a nontumorigenic epithelial cell line (FHs74) were acquired from the American Type Culture Collection (Manassas, VA, USA). Three hundred pairs of surgically resected GC and adjacent noncancerous tissues were obtained from patients who underwent gastrectomy. A freely available integrated dataset (*n* = 1065 GC patients) was accessed at http://kmplot.com/analysis/ [13].

### Transcriptome analysis

The HiSeq System (Illumina, San Diego, CA, USA) was used to perform global expression profiling of 57,749 genes, including splice variants, in clinical specimens (*n* = 4 each) from patients with no metastasis for > 5 years, or patients with peritoneal recurrence, liver recurrence, or distant node metastasis within 2 years after surgery.

### Quantitative reverse-transcription PCR (qRT-PCR) analysis of *NPTXR* and 84 cancer-related genes

Total RNA was extracted from clinical specimens or cell lines using an RNeasy Mini Kit (Qiagen, Hilden, Germany). Specific primers are listed in Additional file 2 (Table S1). Genes expressed in association with *NPTXR* in GC cell lines were analyzed using the Human Epithelial to Mesenchymal Transition RT2 Profiler PCR Array (Qiagen) [14].

### *NPTXR* knockdown (KD), knockout (KO), and overexpression in GC cell lines

To modulate *NPTXR* expression, we generated GC cell lines with small interfering RNA (siRNA)-mediated KD, short hairpin RNA (shRNA)-mediated KD, CRISPR-Cas9-mediated stable KO, and forced overexpression (see Additional file 2: Table S1 1 for sequence details). Genome editing using the CRISPR-Cas9 system was used to generate stable *NPTXR*-KO GC cell lines from MKN1 cells stably expressing luciferase because it expressed one of the highest levels of NPTXR mRNA, had high abilities in cell migration and invasion in our previous studies and is engrafted in nude mice for subcutaneous and peritoneal xenograft models [15, 16].

### Proliferation, apoptosis, and cell cycle assays

Cell proliferation was analyzed using the Cell Counting Kit-8 (CCK-8) assay (Dojindo Molecular Technologies, Inc., Kumamoto, Japan). Apoptosis was measured using an annexin V-Alexa Fluor 568 conjugate (A13202, Thermo Fisher Scientific). Total caspase activity was measured using a Muse Multi-caspase kit (MCH100109, Merck Millipore, Billerica, MA, USA). A Caspase Colorimetric Assay Kit (BioVision, Milpitas, CA, USA) was used to measure the activity of caspase-3, 8, 9, and − 12 individually. The Muse MitoPotential kit (Merck Millipore) was used to assess the mitochondrial membrane potential. Cell cycle progression was analyzed using a Muse Cell Cycle Kit (Merck Millipore). We also analyzed the cell cycle using a Cell Cycle Assay Cell-Clock Kit (Biocolor, Carrickfergus, UK).

### Cell migration, invasion, adhesion and aldehyde dehydrogenase (ALDH) assays

Cell migration was assessed using wound-healing assays. Cell invasion was measured using BioCoat Matrigel invasion chambers (BD Biosciences, Bedford, MA, USA). The CytoSelect 48-Well Cell Adhesion Assay (Cell Biolabs, San Diego, CA, USA) was used to determine cell adhesion to the extracellular matrix proteins. ALDH was measured using the ALDEFLUOR fluorescent reagent system (Stem Cell Technologies, Vancouver, Canada).

### Mouse subcutaneous xenograft model

The Animal Research Committee of Nagoya University approved all animal experiments (approval number 31370). Cells (10^6^/injection) were resuspended in 100 μL of a 1:1 mixture of PBS and Matrigel (BD Biosciences) and injected subcutaneously (s.c.) into both flanks of 9-week-old male BALB/c nu/nu mice (*n* = 3/group; Japan SLC, Inc. Hamamatsu, Japan).

### Generation of rabbit anti-NPTXR pAbs

Two anti-NPTXR pAbs (pAb-1 and pAb-2; antiserum) were generated by immunizing rabbits with synthetic peptides containing NPTXR epitopes predicted to be immunogenic by in silico analysis. Peptides were synthesized by solid-phase peptide synthesis with the fluorenyl-methoxy-carbonyl method (CESGLPRGLQGAGPRRDT for pAb-1 and KERVALSHSSRRQRQEVE for pAb-2).

### Immunofluorescence microscopy and immunohistochemistry (IHC)

Cells were seeded on coverslips and fixed, and then incubated with anti-NPTXR pAb-1 overnight. The cells were washed and then incubated with Alexa Fluor 488-conjugated anti-rabbit IgG (H + L) secondary Ab (Cell Signaling Technology, Danvers, MA, USA).

NPTXR protein expression was analyzed in 80 formalin-fixed and paraffin-embedded sections of well-preserved tissues from patients with GC using anti-NPTXR pAb-1 diluted 1:100 in Ab diluent (Dako, Glostrup, Denmark).

### Generation of mAbs against NPTXR

Three 6-week-old female BALB/c mice were immunized s.c. twice at 3-week intervals with 40 μg of peptide CESGLPRGLQGAGPRRDT and then once more 3 weeks later with 40 μg peptide delivered by intraperitoneal (i.p.) injection. Splenocytes were prepared, fused with P3U1 myeloma cells. The best three clones based on Ab titer and inhibitory activity were selected for further evaluation and designated anti-NPTXR mAb-1, − 2, and − 3. NPTXR mAb-1 was further characterized by epitope mapping and Ab-dependent cell-mediated cytotoxicity (ADCC) assay using an ADCC Reporter Bioassay Kit (Promega, Madison, WI, USA).

### Mouse xenograft model of GC peritoneal metastasis

MKN1 cells stably expressing luciferase (10^6^ cells/mouse) were injected into peritoneal cavity (i.p.) of 10-week-old male BALB/c nu/nu mice (*n* = 4) to establish a model of GC metastasis to the peritoneum. The mice were then injected i.p. twice a week for 6 weeks with 6 μg of normal mouse IgG (control; 140–09511, FUJIFILM Wako Pure Chemical Corporation, Tokyo, Japan), or anti-NPTXR pAb-1, pAb-2, mAb-1, mAb-2, or mAb-3.

### Profiling of intracellular signaling

Phosphorylation of 1006 unique sites in 409 signaling proteins using the PTMScan® Direct Multi-Pathway Kit (Cell Signaling Technology). Pull-down assay was performed to assess activation of small GTPases using an Arf6 Activation Assay Kit and Pan-Ras Activation Assay Kit (STA-407-6 and STA-400, respectively; Cell Biolabs, San Diego, CA, USA).

### Generation of Nptxr^−/−^ mice

*Nptxr*^*−/−*^ mice were generated using the CRISPR/Cas9 system [17]. Mutations in the *Nptxr* allele were confirmed by direct sequencing (Eurofins Genomics Co Ltd., Tokyo, Japan). Appearance and body weight were monitored for 8 weeks, and the development of major organs (macroscopic appearance and histology) and blood tests (blood counts and biochemistry) were evaluated at 8 weeks after birth in groups of *Nptxr*^+/+^, *Nptxr*^+/−^, and *Nptxr*^*−/−*^mice (*n* = 8 each). We also assessed general motor coordination and motor ability using the rotarod test (Economex Rotarod; Columbus Instruments, Columbus, OH, USA) when the mice were 6–8 weeks of age [18]. Three tests were performed (*n* = 10 mice/group) to measure the time spent on the rod, and the longest time was recorded. An arbitrary time limit of 300 s was set for the experiment.

## Results

### Elevated expression of *NPTXR* in GC tissues of patients with peritoneal, hematogenous, and nodal metastasis

We analyzed the transcriptomes of GC tissue specimens from four patients each with no metastasis for > 5 years or with liver, peritoneal, or distant nodal metastasis within 2 years after surgery. An extraction diagram of candidate genes identified by global expression analysis was shown in Additional file 3 (Fig. S1). A total of 14 genes were expressed at significantly higher levels in the 12 patients with metastasis compared to those without (Additional file 4: Table S2). Of these, we selected *NPTXR* for further analysis for three reasons: (i) *NPTXR* encodes a single-pass transmembrane protein that may serve as a target of therapeutic antibodies, (ii) the roles of *NPTXR* in GC have not yet been investigated, and (iii) the nucleotide sequence of *NPTXR* is available (NM_014293.4).

### Expression of *NPTXR* and co-expression of cancer-related genes in GC cell lines

**Fig. 1:**
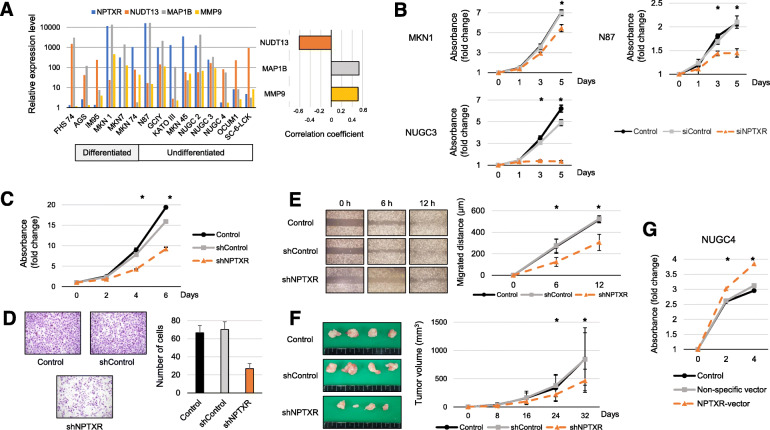
Expression of *NPTXR* in GC cell lines and effect of *NPTXR* knockdown, knockout, or overexpression on cell phenotypes. **a** qRT-PCR analysis of *NPTXR, MAP 1B, MMP9,* and *NUDT13* mRNA in the indicated human GC cell lines. **b** CCK-8 proliferation assay of parental and *NPTXR*-knockdown (KD, siRNA-mediated) MKN1, N87, and NUGC3 cells. **c-f** CCK-8 proliferation assay **c**, wound healing migration assay **d**, Matrigel invasion assay, **e** and in vivo tumorigenicity **f** of parental and *NPTXR*-KD (shRNA-mediated) MKN1 cells. **g** qRT-PCR analysis and CCK-8 proliferation assay of control and *NPTXR*-overexpressing NUGC3 cells. *P < 0.05. Mean ± standard deviation

We performed qRT-PCR analysis of 14 human GC cell lines and found that *NPTXR* mRNA was differentially expressed compared with the control normal epithelial cell line FHs74 (Fig. 1a). Levels of microtubule associated protein 1B (*MAP 1B*) and matrix metallopeptidase 9 (*MMP9*) mRNAs correlated positively with those of *NPTXR* mRNA, whereas there was an inverse correlation between the levels of nudixtype motif 13 (*NUDT13*) and *NPTXR* mRNAs (Fig. 1a).

### Effect of *NPTXR* KD and overexpression on the metastatic behavior of GC cells in vitro

To investigate the involvement of NPTXR in GC cell function, we examined the effects of modulating *NPTXR* levels. MKN1 (differentiated type), N87 (differentiated type), and NUGC3 (undifferentiated type) GC cells were selected for siRNA-mediated and shRNA-mediated *NPTXR* KD based on their relatively high expression levels. We confirmed that si*NPTXR* transfection specifically reduced *NPTXR* mRNA levels in MKN1, N87, and NUGC3 cells compared with the corresponding siControl-transfected cells (Additional file 5: Fig. S2a). In addition, *NPTXR* KD inhibited the proliferation of all three cells lines compared with the siControl and untransfected cells (Fig. 1b). To verify these results, we generated GC cell lines stably expressing *NPTXR* shRNA or a control shRNA. After confirmation of downregulation of *NPTXR* mRNA in sh*NPTXR*-transfected cells compared with the control cells (Additional file 5: Fig. S2b), we examined cell proliferation (Fig. 1c), migration (Fig. 1d), and invasion (Fig. 1e) in vitro*,* and we found that *NPTXR* KD significantly attenuated all three functions. To confirm that these results translated to the growth of GC cells in vivo, we injected shControl- or sh*NPTXR*-expressing MKN1 cells s.c. into BALB/c nu/nu mice and monitored the tumor growth. Indeed, the growth of *NPTXR* KD tumors was reduced compared with that of the control tumors (Fig. 1f).

To determine whether *NPTXR* overexpression had the opposite effects on GC cell behavior, we transfected NUGC4 cells, which expressed low levels of endogenous *NPTXR*, with a control (empty) or *NPTXR* expression plasmid (Additional file 5: Fig. S2c) and examined cell proliferation. Compared with cells expressing the empty plasmid, *NPTXR*-overexpressing NUGC4 cells exhibited enhanced proliferation (Fig. 1g). Thus, *NPTXR* plays a pivotal role in controlling the proliferation and other metastatic behaviors of GC cells.

### CRISPR/Cas9-mediated NPTXR deletion in GC cells

**Fig. 2:**
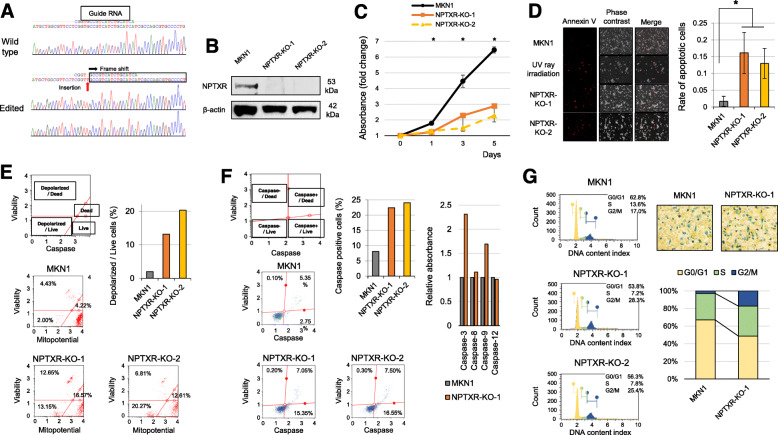
Effects of *NPTXR* knockout on survival phenotypes of GC cell lines. **a** Direct sequence analysis confirming successful editing of *NPTXR* exon 1. Insertion of a T around the target site of the gRNA was observed. Red arrow indicates the frame-shift site. **b** Western blot analysis confirming *NPTXR* knockout (KO) in MKN1 cells. **c-g** CCK-8 proliferation assay **c**, annexin V flow cytometric apoptosis assay **d**, flow cytometric mitochondrial membrane potential assay **e**, colorimetric activated caspase-3 and -9 assay **f**, and flow cytometric cell cycle analysis assay **g** of parental and *NPTXR* KO MKN1 cells. *P < 0.05. Mean ± standard deviation

We selected MKN1 cells for *NPTXR* KO because it expressed high *NPTXR* expression levels and was originally derived from a liver metastasis lesion from a GC patient. After CRISPR/Cas9 editing, two clones with stable *NPTXR* KO were generated (KO-1 and KO-2), and confirmed by direct sequence analysis to harbor the expected deletion (Fig. 2a). NPTXR protein was also undetectable in both clones by western blot analysis (Fig. 2b).

### Effect of stable NPTXR KO on the survival phenotype of GC cell lines

To further investigate the oncological functions of *NPTXR*, we examined the proliferation, cell cycle progression, and apoptosis of KO-1, KO-2, and parental MKN1 cells. As expected from the results with *NPTXR* KD cells, the proliferation of KO-1 and KO-2 cells was attenuated compared with the control cells (Fig. 2c). In addition, apoptosis was significantly more prevalent among the KO cell lines than the parental line, as determined by annexin V staining (Fig. 2d). To determine whether apoptosis in *NPTXR* KO cells occurred via the mitochondrial pathway, we also evaluated differences in mitochondrial membrane potential using a fluorescence assay. Notably, a higher proportion of KO-1 and KO-2 cells than control cells exhibited disruption of the mitochondrial membrane potential (Fig. 2e). Moreover, total caspase activity was increased in KO-1 and KO-2 cells compared with control MKN1 cells (Fig. 2f), with preferential increases detected in caspase-3 and -9 activities (Fig. 2f). To evaluate the effects of *NPTXR* KO on cell cycle progression, we examined the proportion of cells in each phase of the cycle. *NPTXR* KO resulted in an accumulation of cells in G2/M phase compared with control MKN1 cells, as measured using both flow cytometric and colorimetric cell cycle assays (Fig. 2g).

### Effects of stable NPTXR KO on the metastatic potential phenotype of GC cells

**Fig. 3:**
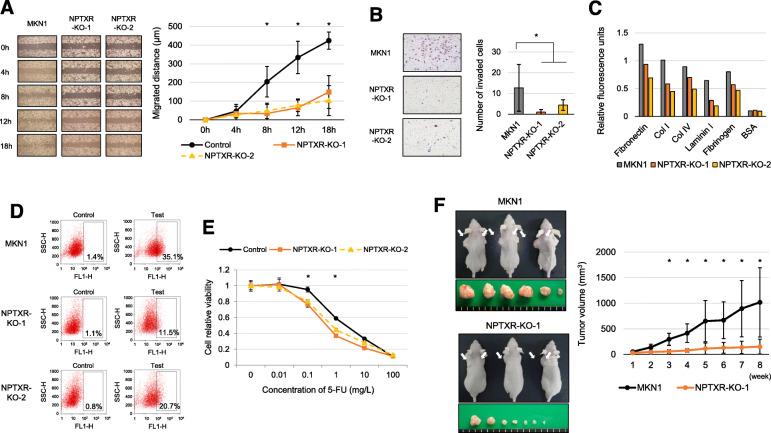
Effects of *NPTXR* knockout on the carcinogenic and drug resistance phenotypes of GC cell lines. **a-c** Wound-healing migration assay **a**, Matrigel invasion assay **b**, and extracellular matrix protein adhesion assay **c** of parental, *NPTXR*-KO-1, and *NPTXR*-KO-2 MKN1 cells. **d** Flow cytometric assay of ALDH-positive control (left panels) and *NPTXR*-KO (test, right panels) MKN1 cells. **e** CCK-8 proliferation assay of 5-fluorouracil (FU) sensitivity of parental and *NPTXR*-KO MKN1 cells after 3 days incubation. **f** Growth of parental and *NPTXR-*KO MKN1 xenografts in BALB/c nu/nu mice. *P < 0.05. Mean ± standard deviation

Next, we evaluated several characteristic oncological behaviors in *NPTXR* KO and control MKN1 cells. Both migration, as measured using a wound-healing assay (Fig. 3a), and invasion, as measured using Matrigel assays (Fig. 3b) were attenuated by *NPTXR* KO in both cell lines compared with control MKN1 cells. In addition, the KO cells exhibited reduced adhesion to five ECM proteins compared with control MKN1 cells (Fig. 3c). To determine whether *NPTXR* KO affects the presence of GC subpopulations with cancer stem cell-like properties, we examined ALDH levels and found that ALDH was reduced in the KO-1 and KO-2 cells compared with the control MKN1 cells (Fig. 3d). Consistent with the essential role of *NPTXR* in GC cell biology detected here, we also found that *NPTXR* KO reduced the sensitivity of MKN1 cells to 5-FU, as measured by cell proliferation (Fig. 3e). Finally, we confirmed the influence of stable *NPTXR* KO on tumor growth in vivo using the BALB/c nu/nu mouse xenograft model, in which control or *NPTXR*-KO-1 MKN1 cells were injected s.c. While tumors formed by the control cells grew progressively over eight weeks, *NPTXR* KO greatly reduced cell growth (Fig. 3f), as indicated by the significantly smaller tumor volumes formed by *NPTXR*-KO-1 cells compared with the control MKN1 cells (Fig. 3f).

### Production of pAbs against NPTXR and efficacy in vivo

**Fig. 4:**
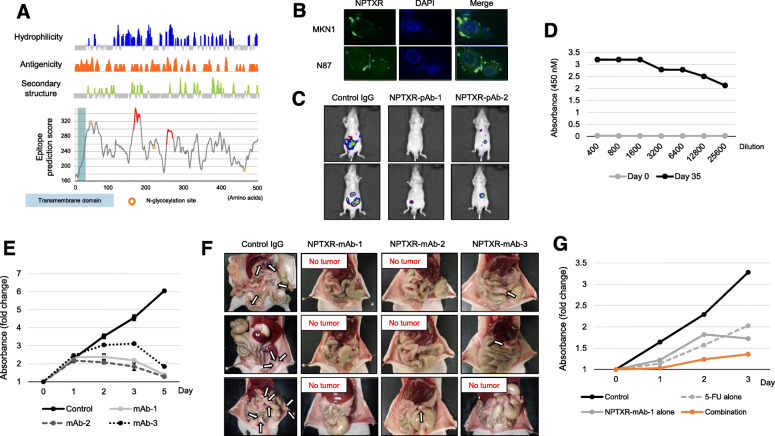
Characterization of anti-NPTXR pAbs and mAbs. **a** Bioinformatics analysis of predicted immunogenic epitopes in *NPTXR*. **b** Immunofluorescence microscopy of MKN1 and N87 cells labeled with NPTXR-pAb-1 and Alexa Fluor 488 (green). Nuclei were stained with DAPI (blue). **c** IVIS analysis of representative BALB/c nu/nu mice injected with luciferase-expressing parental or *NPTXR*-KO MKN1 cells. Mice were injected with d-luciferin for 15 min and imaged 3 weeks after cell injection. **d** ELISA assay of sera from mice 3 weeks after immunization and boosting with NPTXR peptide. **e** CCK-8 proliferation assay of MKN1 cells incubated with NPTXR mAb-1, − 2 and − 3. **f** Therapeutic effects of intraperitoneal administration of anti-NPTXR monoclonal antibodies. **g** CCK-8 proliferation assay of MKN1 cells treated with 5-fluorouracil (5-FU) and NPTXR-mAb-1. *P < 0.05. Mean ± standard deviation

To assess the potential anti-cancer therapeutic value of Ab-mediated blockade of NPTXR in GC and to determine the optimal antigenic site, we next generated and tested pAbs. Rabbits were immunized with two NPTXR peptides predicted to be immunogenic (Fig. 4a). Antisera were collected and two preparations of pAbs were purified and designated pAb-1 and pAb-2. The pAbs were then used to examine the subcellular localization of NPTXR by immunofluorescence microscopy. In both MKN1 and N87 cells, NPTXR was detected at the plasma membrane, as expected from its known structure (Fig. 4b). We also determined whether pAb-mediated blockade of NPTXR could attenuate GC cell proliferation, and found that both pAb-1 and pAb-2 significantly inhibited the proliferation of MKN1 cells compared with IgG control treatment (Additional file 6: Fig. S3a).

We next evaluated the potential therapeutic effects of the NPTXR pAbs in vivo. Whole-body imaging of the mice revealed an intense luciferin fluorescence signal in mice injected with control IgG but a much reduced signal in animals in the pAb-1 and pAb-2 treatment groups three weeks after cell injection (Fig. 4c). The macroscopic appearance of peritoneal metastases 6 weeks after cell injection is shown in Additional file 6 (Fig. S3b). Few peritoneal nodules were observed in the mice treated with anti-NPTXR pAbs, whereas many large tumor nodules were present in the omentum and mesenteric tissue in mice treated with control IgG (Additional file 6: Fig. S3b). Consistent with this, the total volume of peritoneal nodules was significantly smaller in mice treated with pAbs compared with control IgG (Additional file 6: Fig. S3b). Importantly, over the 6-week experiment, anti-NPTXR pAbs had no significant effect on gross clinical signs, food intake, or body weight. These results that Ab-mediated blockade of NPTXR may be a feasible approach to the treatment of GC.

### Production and characterization of mAbs against NPTXR

Having established that anti-NPTXR pAbs suppress tumor growth in vitro and in vivo, we next generated mouse mAbs against the same NPTXR sequence (161–178 CESGLPRGLQGAGPRRDT) as that used to raise pAb-1. After fusion of splenocytes and screening and cloning of hybridomas, we selected three mAb-producing clones for expansion and purification of mAbs (designated mAb-1, − 2, and − 3; Fig. 4d and Fig. 4e). The therapeutic potential of the three mAbs was assessed using the mouse xenograft model. BALB/c nu/nu mice were injected i.p. with parental MKN1 cells and then injected i.p. with 6 μg of control IgG or mAb-1, − 2, or − 3 twice weekly for 4 weeks. The macroscopic appearance of peritoneal metastases 4 weeks after cell injection is shown in Fig. 4f. Tumor nodules were observed in the omentum and mesenteric tissue of all mice treated with control IgG, whereas none were found in the mAb-treated mice. As expected from this observation, the total volume of peritoneal nodules was smaller for the groups treated with mAb-1, mAb-2, and mAb-3 groups compared with the control IgG group (Additional file 6: Fig. S3c). No overt signs of mAb toxicity were observed in any of the mice over the 4-week treatment period.

Based on these findings, we selected NPTXR-mAb-1 for further characterization. mAb-1 inhibited the proliferation of a range of GC cell lines of differential histologic types and with different NPTXR expression levels (Additional file 7: Fig. S4a), including N87 (differentiated) and MKN45 (undifferentiated) cells. To determine whether mAb-1 treatment affects drug resistance, MKN1 cells were incubated with mAb-1 in combination with various concentrations of 5-FU or cisplatin. Notably, mAb-1 synergistically enhanced the antiproliferative effect of both 5-FU (Fig. 4g) and cisplatin (Additional file 7: Fig. S4b), consistent with the finding that *NPTXR* KO sensitized MKN1 cells to 5-FU.

**Fig. 5:**
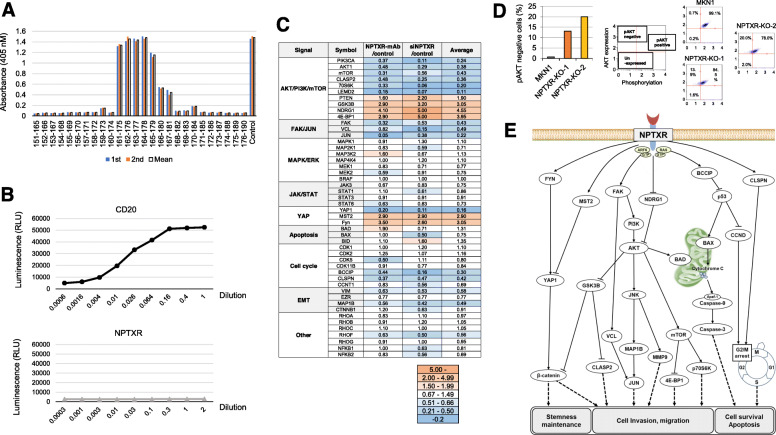
Signal transduction pathways associated with NPTXR. **a** Epitope mapping of the amino acid sequence targeted by NPTXR-mAb-1 by ELISA. **b** Antibody-dependent cell-mediated cytotoxicity assay of MKN1 cells in the presence of CD20-mAb or NPTXR-mAb-1. **c** Overview of the antibody array results. Data are expressed as the fold-change induced by treatment of MKN1 cells with NPTXR-mAb-1 or by transfection with NPTXR-targeting siRNA. **d** Analysis of phosphorylated AKT levels in MKN1 or *NPTXR*-KO cells. **e** Hypothetical role of *NPTXR* in signaling pathways and cell phenotypes

To determine the precise epitope in NPTXR recognized by mAb-1, we performed epitope mapping using a competition ELISA that tested 26 peptides (15-mers) encompassing amino acids 151–190 of NPTXR (IRELTGKLGRCESGLPRGLQGAGPRRDTMADGPWDSPALI). This analysis identified GLPRGLQGAGPRRDT as the epitope recognized by mAb-1 (Fig. 5a). Next, we probed the potential anti-cancer mechanism of action of anti-NPTXR mAb-1. In addition to steric or functional blockade of target proteins, therapeutic Abs can target virus-infected or other diseased cells for destruction by immune cells, such as natural killer cells, through the process of ADCC. Using a cell-based ADCC reporter assay, we found that NPTXR-mAb-1 could not mediate ADCC (Fig. 5b), suggesting that its in vivo ability to reduce tumor growth occurred via a non-cytolytic mechanism.

### Association of NPTXR with intracellular signaling pathways

NPTXR-KO-1 and NPTXR-KO-2 cells expressed lower levels of MAP 1B and MMP9 and higher levels of NUDT13 compared with those of MKN1 cells (Additional file 8: Fig. S5a), supporting the reproducibility of the PCR array analysis. We identified signals altered both in cells treated by siNPTXR and NPTXR-mAb using the antibody array analysis, and the results were summarized in Fig. 5c*.* Loss of *NPTXR* function suppressed the phosphorylation of PI3K and AKT, enhanced the phosphorylation of GSK3B, and suppressed signaling via the FAK–JNK, YAP, and β-catenin pathways. In contrast, *NPTXR* inhibition or silencing had no effect on the phosphorylation of JAK or BCL-2, or on MEK–p44 MAPK–ERK1/2 signaling. With respect to signaling events involved in cell cycle regulation, phosphorylation of BCCIP, CLSPN, and CCND was decreased by inhibition/silencing of *NPTXR*. The reproducibility of the findings from the antibody array experiments was assessed by flow cytometric analysis of MAPK and PI3K signaling. *NPTXR* KO had no effect on the phosphorylation of ERK in either of the KO cells (Additional file 8: Fig. S5b), but phosphorylation of AKT (a surrogate marker of activation of the PI3K pathway) was reduced compared with control MKN1 cells (Fig. 5d). Moreover, we found that the small GTPases RAS-GTP and Arf6-GTP were inactivated by *NPTXR* KO, suggesting that they may be potential scaffolding proteins of *NPTXR* (Additional file 8: Fig. S5c). Collectively, these data suggest that *NPTXR* plays numerous roles in intracellular dynamics and signaling pathways, as summarized in Fig. 5e.

### Generation and characterization of Nptxr^−/−^ mice

**Fig. 6:**
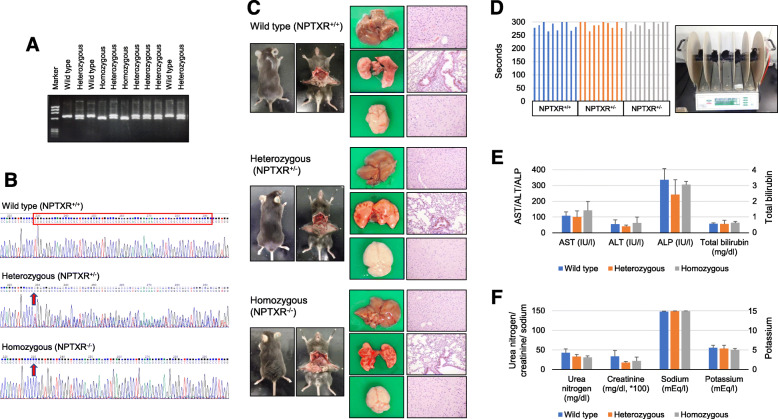
Characterization of *Nptxr*^*−/−*^ mice. **a** Genotyping of *Nptxr*^+/+^, *Nptxr*^+/−^, and *Nptxr*^*−/−*^ mice. **b** Direct sequencing of the 64-nucleotide deletion at the *NPTXR* locus of *Nptxr*^*−/−*^ mice. **c** Appearance of major organs from *Nptxr*^+/+^, *Nptxr*^+/−^, and *Nptxr* ^*−/−*^ mice. **d** Rotarod test of motor coordination and learning by *Nptxr*^+/+^, *Nptxr*^+/−^, and *Nptxr* ^*−/−*^ mice. **e** and **f** Renal **e** and liver **f** function tests of *Nptxr*^+/+^, *Nptxr*^+/−^, and *Nptxr* ^*−/−*^ mice

To clarify the pathophysiological functions of *NPTXR*, we generated *Nptxr*^+/+^, *Nptxr* ^+/−^_,_ and *Nptxr* ^*−/−*^ mice (Fig. 6a). Direct sequencing analysis of the mice confirmed the deletion of 64 bases by genome editing at the *Nptxr* locus (Fig. 6b). KO of one or both *Nptxr* alleles (*Nptxr*^*+/−*^ and *Nptxr*^*−/−*^ mice) was not embryonic lethal and had no effect on the appearance (Fig. 6c) or body weight (Additional file 9: Fig. S6a) of the mice, and did not result in any abnormalities in the development of the liver, lung, and brain (Fig. 6c), metabolic parameters (Additional file 9: Fig. S6b), or hematological parameters (Additional file 9: Fig. S6c). Moreover, neither the *Nptxr*^*+/−*^ nor the *Nptxr*^*−/−*^ mice exhibited dysfunctional motor coordination or motor learning, as measured by the rotarod test (Fig. 6d).

### Clinical significance of *NPTXR* expression in GC tissues

**Fig. 7:**
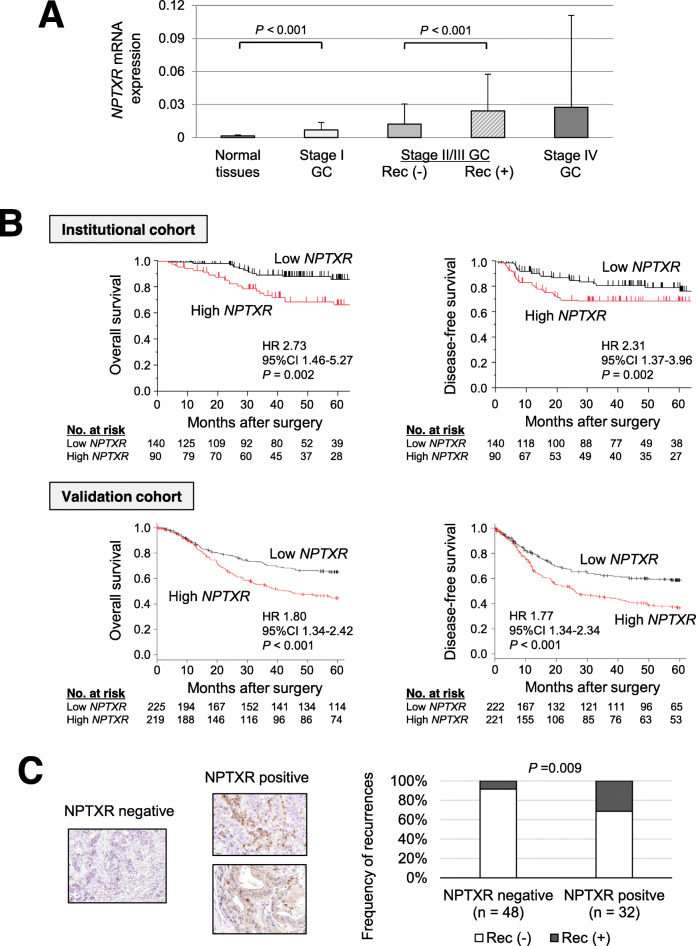
Clinical significance of *NPTXR* expression in gastric tissues from patients with GC. **a** qRT-PCR analysis of *NPTXR* mRNA levels in 80 pairs of GC and matched adjacent normal gastric tissues according to disease stage and the presence or absence of disease recurrence (Rec). **b** Kaplan–Meier curves of overall and disease-free survival of patients in the institutional and validation cohorts with stage I–III GC according to *NPTXR* expression. **c** Representative images of IHC staining of NPTXR protein expression in gastric tissue sections. Left panel, section with undetectable NPTXR expression; right panels, sections with positive NPTXR expression

*NPTXR* mRNA levels increased incrementally in specimens from patients with stage I, II/III, and IV GC compared with normal gastric mucosal tissue (Fig. 7a). Patients with stage II/III GC who developed disease recurrence had significantly higher *NPTXR* mRNA levels compared with patients without recurrence. In ROC curve analysis of the ability of *NPTXR* levels to predict cancer-related mortality, the AUC was 0.651 and the optimal cut-off value was 0.0086 (Additional file 10: Fig. S7a). Using this cut-off value, patients were dichotomized into high- or low-*NPTXR* expression groups. We found that high *NPTXR* expression was significantly associated with male sex, macroscopic Borrmann type 4 or 5, pathological T4, and undifferentiated tumor type (Additional file 11: Table S3). We next focused on 230 patients with stage I–III GC and analyzed survival after curative resection. After dichotomization of patients using the ROC cut-off value, the high-*NPTXR* group was found to have shorter overall and disease-free survival times than the low-*NPTXR* group (Fig. 7b). Similar results were obtained using an external validation dataset of 1065 patients (Fig. 7b). Multivariable analysis identified high *NPTXR* expression as an independent prognostic factor for GC patients (Additional file 12: Table S4). The high-*NPTXR* group also had a higher prevalence of peritoneal, lymph node, and hematogenous recurrence compared with the low-*NPTXR* group (16% vs 6, 13% vs 6, and 11% vs 8%, respectively) (Additional file 10: Fig. S7b). We conducted a subgroup analysis according to postoperative adjuvant chemotherapy (5-FU-based) to explore further the clinical significance of *NPTXR* expression in GC. Influence of the *NPTXR* expression on survival depended on whether the patients received postoperative adjuvant chemotherapy or not. The survival difference between the low and high *NPTXR* groups was greater in the subgroup of patients who received adjuvant chemotherapy after curative gastrectomy compared to that of patients who underwent surgery alone (Additional file 10: Fig. S7c). These results suggest that *NPTXR* expression reflects resistance to adjuvant chemotherapy.

### IHC analysis of NPTXR expression in gastric tissues

Finally, we explored the utility of the anti-NPTXR pAb-1 as a potential diagnostic tool by performing IHC staining of GC tissues. Images of representative sections with negative or positive NPTXR immunoreactivity are shown in Fig. 7c. Among the 80 patients assessed, 32 were positive for NPTXR in the primary tumor, and the incidence of disease recurrence was higher in patients with NPTXR-positive tumors compared with those with NPTXR-negative tumors (31 and 8%, respectively, *P* = 0.009; Fig. 7c).

## Discussion

Metastasis is a major cause of death in patients with GC [1, 19]. A better understanding of the molecular mechanisms underlying metastasis is therefore essential for developing novel and innovative therapeutic strategies and risk assessment biomarkers [20, 21]. As a first step towards this, we conducted transcriptome analysis of GC tissues from patients with and without metastasis and identified *NPTXR* as one of 14 genes with significant overexpression in GC tissues metastasized to diverse organs. We generated GC cell lines with *NPTXR* KD, KO, and overexpression, developed anti-NPTXR pAbs and mAbs, investigated the involvement of *NPTXR* in GC cell functions in vitro and in vivo, and generated a new strain of *Nptxr*^*−/−*^ mice. Our results indicate that *NPTXR* regulates several cell behaviors associated with the malignant phenotype and metastatic potential of GC cells via multiple intracellular signaling pathways, including the PI3K–AKT–mTOR and FAK–JNK pathways. We also showed that anti-NPTXR pAb and mAbs had therapeutic effects in a mouse model of peritoneal metastasis. It is a significant advance in further understanding of physiological roles of NPTXR that we first found no adverse effects on reproduction, development, or metabolic or motor functions by genetic deletion of *NPTXR* in mice. Collectively, these results not only suggest that *NPTXR* may be an excellent therapeutic target for metastatic GC but also indicate that NPTXR-targeting mAbs can effectively suppress GC growth in vivo in the absence of ADCC.

*NPTXR* is a receptor for synapse-associated neuronal NPTX, and it is expressed at higher levels in the brain compared with other organs [11, 22]. Among the NPTX family proteins, *NPTXR* is unique in possessing a transmembrane domain and in its ability to anchor *NPTX* complexes to plasma membranes [23, 24]. *NPTXR* is also cleaved by the extracellular protease tumor necrosis factor-α converting enzyme to release a soluble form of *NPTXR* [10, 25]. Members of the NPTX family function during synapse formation and remodeling by facilitating the uptake of synaptic materials [24, 25]. *NPTXR* and *NPTX2* are overexpressed in human Schwannian stroma-poor neuroblastoma [12, 22, 26], and *NPTX2* expression serves as a marker of poor prognosis of neuroblastoma patients [12]. Bartolini et al. investigated expression and functions of *NPTX1*, *NPTX2* and *NPTXR* in glioblastoma [12]. They found that blockade of ligand-receptor connection between *NPTX2* and *NPTXR* reduces tumor burden in orthotopic mouse models of human neuroblastoma [12]. However, the oncological roles of *NPTXR* in gastroenterological malignancies are unknown.

We focused on determining the involvement of *NPTXR* in the malignant phenotype of GC cells by gene-specific ablation or overexpression and Ab-mediated inhibition of function. *NPTXR* KD, KO, or Ab-mediated blockade significantly inhibited the proliferation of multiple GC cell lines, possibly via facilitation of apoptosis and/or induction of G2/M arrest [27]. Apoptosis is predominantly mediated via two pathways; the intrinsic mitochondria pathway, which involves activation of caspases and the disruption of mitochondrial membrane potential, and the extrinsic death receptor pathway, which requires engagement of cell surface death receptors [28, 29]. Notably, *NPTXR* KO increased caspase activity, particularly caspase-9, and induced a striking decrease in mitochondrial membrane potential. Caspase-9 is a cysteine-aspartic protease associated with cytokine signaling and apoptosis [30, 31]. Apoptotic signals cause the release of cytochrome c from mitochondria and activation of apoptosis protease-activating factor 1, which then cleaves procaspase-9 into the active homodimer [30, 31]. Our results thus suggest that NPTXR regulates apoptosis through mitochondrial-dependent activation of caspase-9.

The involvement of *NPTXR* in oncogenesis is also supported by our findings that *NPTXR* KO attenuated many biological properties (migration, invasion, and stemness) required for GC metastasis. High ALDH activity is a hallmark of cancer stem cells [32], which are able to evade the effects of chemotherapy and radiotherapy through their ability to self-renew and differentiate into tumor cells after treatment is discontinued, leading to recurrence and/or metastasis [33]. In the present study, *NPTXR* KO decreased the frequency of ALDH-positive GC cells, indicating that *NPTXR* influences the stemness of GC cells. We speculate that the decreased stemness of *NPTXR*-KO cells may be associated with their increased sensitivity to 5-FU. *NPTXR* KO also resulted in a reduction of tumor growth in the mouse xenograft model of s.c. GC.

Consistent with the paucity of information on the roles of *NPTXR* in oncogenesis, little is known about the interactions between *NPTXR* and cancer-related pathways. We explored NPTXR-associated gene expression using two approaches. First, we performed PCR array analysis, which showed that *NPTXR* expression may influence or be influenced by *MAP 1B*, *MMP9*, and *NUDT13*, which contribute to the EMT, a crucial process in carcinogenesis. *MAP 1B* is upregulated in cells during an EMT-like process that operates during wound healing [34, 35]. *MMP9* is a matrix metalloproteinase involved in the degradation of the ECM in physiological processes such as tissue remodeling and embryonic development, as well as in pathological processes such as the progression of arthritis and cancer metastasis [36, 37]. *NUDT13* is a mitochondrial enzyme reported to be downregulated during the EMT [38]. As a second method to explore cancer-related pathways, we performed antibody array analysis to identify alterations in the phosphorylation of intracellular signaling proteins GC cells with loss of *NPTXR* expression or function. *NPTXR* KO or mAb treatment resulted in inactivation of the PI3K–AKT–GSK3B, FAK–JNK, and YAP signaling pathways, but not of MAPK and JAK–STAT signaling. PI3K–AKT–mTOR is a key signaling pathway involved in numerous cellular processes [39]; YAP1 modulates cancer stem cell properties, such as sphere formation, self-renewal, invasiveness, and drug resistance, via the Hippo and Wnt/β-catenin pathways [40]; and FAK regulates cell growth, survival, migration, and invasion through its dual functions as a kinase and a scaffold protein [41]. FAK autophosphorylation leads to the recruitment of Src and the phosphorylation of binding sites for downstream effector pathways, including JNK and the transcriptional regulation of pro-invasive genes like MMP9 [41, 42]. Previous studies indicate that the PI3K–AKT–GSK3B, FAK–JNK, and YAP signaling pathways interact with each other during cancer development and progression. Kinase function of FAK has been shown to activate the PI3K-AKT pathway [43]. FAK binds p85 subunit of PI3K, and phospholipid production stimulated by FAK association and activation of PI3K can activate AKT kinase, leading to inhibition of apoptosis by regulating various cell death machinery proteins [43, 44]. Moreover, it has been reported that concomitant activation of YAP1 and PI3K promotes development of liver tumors through activation of AKT/mTOR signaling [40]. Our findings suggest that NPTXR modulates the network of AKT, FAK and YAP pathways and contributes to GC progression.

We investigated the potential therapeutic effects of anti-NPTXR Abs using a mouse xenograft model of peritoneal metastasis. Both pAbs and mAbs significantly inhibited tumor formation in this model and the anti-NPTXR mAbs also enhanced the cytotoxicity of 5-FU and cisplatin in vitro, supporting the possibility that NPTXR may be a promising therapeutic target for controlling GC progression and/or for overcoming chemotherapy resistance. We also showed that the anti-cancer effects of the mAbs in vivo resulted from NPTXR antagonism rather than as mediators of ADCC [45]. Finally, we identified the precise epitope recognized by our NPTXR mAbs, which is a critical step in developing and engineering therapeutic mAbs. Further work, including humanization of the mAbs and additional preclinical efficacy and toxicity testing, will be required before these promising findings can be translated to the clinic. Along these lines, another important finding in the present study is that *Nptxr*^*−/−*^ mice exhibit no abnormalities in reproduction, development, metabolism, or motor functions, and that treatment of mice for up to 6 weeks with anti-NPTXR pAbs and mAbs elicited no overt clinical signs or adverse effects. These are important observations for understanding the potential effects of clinical *NPTXR* targeting on physiological homeostasis.

Our findings have clinical relevance, as demonstrated by the association between *NPTXR* expression in patient specimens and outcomes. We found that *NPTXR* mRNA level in clinical samples is an independent prognostic factor in GC, and these findings were confirmed using an independent external validation dataset. Our IHC analysis also indicated that NPTXR protein expression may serve as a novel biomarker of disease recurrence, which can easily be determined through analysis of surgical specimens or endoscopic biopsy samples. Further, like HER2 amplification *NPTXR* expression may be utilized, for example, as a companion diagnostic to identify patients potentially sensitive to anti-*NPTXR* therapy.

Several limitations of our study should be acknowledged. It remains unclear whether NPTX1 and NPTX2 act as ligands of NPTXR in GC cells or not. We evaluated the effects of anti-*NPTXR* pAbs and mAbs only in a mouse model, and the frequency, administration route, optimal dose, and optimal duration of treatment and survival benefits were not determined. Patient-derived xenograft models may be helpful for understanding the oncological involvement of *NPTXR*. The development of assays to detect NPTXR expression levels in serum will likely facilitate the translation of our findings to the clinic.

## Conclusions

We identified *NPTXR* as a cell surface receptor that modulates multiple oncological processes in GC cells, including behaviors associated with metastasis. NPTXR-targeting Abs may have utility in GC, not only as innovative therapeutic agents but also as companion diagnostic tools.

## Supplementary information

**Additional file 1.** Supplementary Materials and Methods.

**Additional file 2: Table S1.** Nucleotide sequences.

**Additional file 3: Figure S1.** Extraction diagram of candidate genes identified by global expression analysis. Sixteen patients were categorized into four groups (4 patients for each) according to clinical courses as follows: group 1, no recurrences for > 5 years; group 2, hepatic-confined metastasis within 2 years after surgery; group 3, peritoneal metastasis within 2 years after surgery; and group 4, nodal metastasis within 2 years after surgery. Global expression profiling was conducted to compare the expression levels of 57,749 genes between each cluster. Among them, 14 we considered 14 genes as candidates shown in Supplemental Table 2.

**Additional file 4: Table S2.** List of candidate genes upregulated in gastric cancer tissues of patients with metachronous metastasis.

**Additional file 5: Figure S2.** Verification of *NPTXR* knockdown or overexpression in GC cells. a-c qRT-PCR analysis of *NPTXR* mRNA expression in MKN1, N87, and/or NUGC3 cells with siRNA-mediated knockdown (a), shRNA-mediated knockdown (b), or overexpression (c) of *NPTXR*. *P < 0.05. Mean ± standard deviation.

**Additional file 6: Figure S3.** Characterization of polyclonal anti-NPTXR Abs effects in vitro and in vivo. a *CCK-8* proliferation assay of MKN1 cells incubated with pAbs in vitro. b Therapeutic effects of intraperitoneal administration of anti-NPTXR polyclonal antibodies*.* Macroscopic appearance of peritoneal nodules 6 weeks after injection of MKN1 cells and treatment with control IgG or NPTXR-pAbs. c Total volumes of peritoneal nodules collected from BALB/c nu/nu mice 6 weeks after injection of MKN1 cells and treatment with control IgG or NPTXR-mAb-1, − 2, or − 3. *P < 0.05. Mean ± standard deviation.

**Additional file 7: Figure S4.** Effects of NPTXR-mAb-1 on gastric cancer cells. CCK-8 proliferation assay of MKN45 (a) and N87 (b) cells incubated with NPTXR-mAb-1 in dose-dependent manner.

**Additional file 8: Figure S5.** a qRT-PCR analysis of *NUDT13, MAP 1B, MMP9* mRNA levels in parental MKN1, *NPTXR*-KO-1 and -KO-2 cells. b Flow cytometric analysis of the frequency parental and *NPTXR*-KO MKN1 cells with phosphorylated ERK1/2. c Pull-down of small GTPases (Ras and Arf6) in parental and *NPTXR*-KO MKN1 cells.

**Additional file 9: Figure S6.** a Body weights of *Nptxr*^+/+^, *Nptxr*^+/−^, and *Nptxr*^*−/−*^ mice at 4 and 8 weeks after birth. b and c Metabolic (b) and hematological (c) tests in *Nptxr*^+/+^, *Nptxr*^+/−^, and *Nptxr*^*−/−*^ mice.

**Additional file 10: Figure S7.** a ROC curve analysis of the ability of *NPTXR* expression level in tissue specimens to predict peritoneal metastasis in GC patients. b Frequency of the site of initial recurrence in GC patients according to *NPTXR* expression level. c Disease-free survival rates in subgroups according to administration of adjuvant chemotherapy.

**Additional file 11: Table S3.** Patients’ clinical characteristics associated with *NPTXR* expression.

**Additional file 12: Table S4.** Prognostic factors of patients with resectable gastric cancer.

## Data Availability

All the data obtained and/or analyzed during the current study were available from the corresponding authors on reasonable request.

## Abbreviations

ALDH: Aldehyde dehydrogenase
AUC: Area under the curve
IHC: Immunohistochemistry
NPTXR: Neuronal pentraxin receptor
qRT-PCR: Quantitative reversetranscription polymerase chain reaction
ROC: Receiver operating characteristic
siRNA: Small interfering RNA
