# Supplementary material for: Therapeutic monoclonal antibody targeting of neuronal pentraxin receptor to control metastasis in gastric cancer
Source: Mol Cancer. 2020 Aug 26;19:131. doi: 10.1186/s12943-020-01251-0 (PMC7448342; doi:10.1186/s12943-020-01251-0)
Supplement: Supplementary file 1 — Additional file 1. Supplementary Materials and Methods. [file 12943_2020_1251_MOESM1_ESM.docx]

**Therapeutic monoclonal antibody targeting of neuronal pentraxin receptor to control metastasis in gastric cancer**

Mitsuro Kanda, et al.

**Supplemental Materials and Methods**

*Cell Lines and Clinical Samples*

The GC cell lines GCIY, IM95, MKN1, MKN7, MKN45, MKN74, NUGC2, NUGC3, NUGC4, OCUM-1, and SC-6-JCK were obtained from the Japanese Collection of Research Bioresources Cell Bank (JCRB, Osaka, Japan). AGS, KATOIII, and N87 GC cell lines and a nontumorigenic epithelial cell line (FHs74) were acquired from the American Type Culture Collection (Manassas, VA, USA). All cell lines were authenticated using the short tandem-repeat polymerase chain reaction (PCR) method by the JCRB Cell Bank before the study commenced. Three hundred pairs of surgically resected GC and adjacent noncancerous tissues were obtained from patients who underwent gastrectomy at the Department of Gastroenterological Surgery, Nagoya University Hospital between 2001 and 2017. The isolated tissues were frozen in liquid nitrogen and stored at −80°C until analysis. The Institutional Review Board of Nagoya University, Japan, approved this study, and written informed consent was obtained from all patients. A freely available integrated dataset (n = 1065 GC patients) compiled by three major cancer research centers (Berlin, Bethesda, and Melbourne datasets) was accessed at http://kmplot.com/analysis/.[[1](#_ENREF_1)] This dataset was used to validate the results obtained with our institutional dataset on the prognostic significance of tumor *NPTXR* expression.

*Transcriptome Analysis*

The HiSeq System (Illumina, San Diego, CA, USA) was used to perform global expression profiling of 57,749 genes, including splice variants, in clinical specimens (n = 4 each) from patients with no metastasis for >5 years, or patients with peritoneal recurrence, liver recurrence, or distant node metastasis within 2 years after surgery.

*Quantitative Reverse-Transcription PCR (qRT-PCR) Analysis of NPTXR and 84 Cancer-Related Genes*

Total RNA was extracted from clinical specimens or cell lines using an RNeasy Mini Kit (Qiagen, Hilden, Germany). Real-time PCR was performed using SYBR Green and an ABI StepOnePlus Real-Time PCR System (Applied Biosystems, Foster City, CA). Cell lines were analyzed as biological triplicates and both tissues and cell lines were analyzed as technical triplicates. Glyceraldehyde-3-phosphate dehydrogenase (*GAPDH*) mRNA was measured as an endogenous control. Specific primers are listed in Supplementary Table 1. Genes expressed in association with *NPTXR* in GC cell lines were analyzed using the Human Epithelial to Mesenchymal Transition RT2 Profiler PCR Array (Qiagen). This kit detects 84 key genes with functions in transcription, extracellular matrix (ECM) formation, epithelial-to-mesenchymal transition (EMT), differentiation, morphogenesis, growth, proliferation, migration, and cytoskeletal formation.[[2](#_ENREF_2)]

*NPTXR Knockdown (KD), Knockout (KO), and Overexpression in GC Cell Lines*

To modulate *NPTXR* expression, we generated GC cell lines with small interfering RNA (siRNA)-mediated KD, short hairpin RNA (shRNA)-mediated KD, CRISPR-Cas9-mediated stable KO, and forced overexpression (see Supplementary Table 1 for sequence details). For siRNA-mediated KD, MKN1, N87, and NUGC3 cells were cultured at 5 × 10^4^/well in a 24-well plate and transiently transfected with 30 nM of si*NPTXR* or control siRNA (siControl) for 72 h using the Neon Transfection System (Thermo Fisher Scientific, Waltham, MA, USA) according to the manufacturer’s recommendations. shRNA-mediated knockdown was performed using MISSION® shRNA Lentiviral Transduction Particles (SHCLNV-NM_014293, Merck) according to the manufacturer’s protocol. MKN1 cells (1.5 × 10^3^) were incubated in a 96-well plate for 24 h, and sh*NPTXR* or shControl lentiviral particles (pLKO.1-puro, SHC001V, Merck; 4 μL/well) and hexadimethrine bromide (final concentration 8 μg/mL) were added to the wells. The plates were incubated for 8 h, and puromycin (1 μg/mL) was added to the medium to select for shRNA-expressing cells. For *NPTXR* overexpression, 3 × 10^3^ NUGC4 cells were transfected with an *NPTXR* expression vector (RC212087; OriGene Technologies, Rockville, MD, USA) or control empty vector (PS100001; OriGene Technologies) by electroporation using the Neon system (Thermo Fisher Scientific) and incubated for 48 h.

Genome editing using the CRISPR-Cas9 system was used to generate stable *NPTXR*-KO GC cell lines.[[3](#_ENREF_3)] Guide RNA (gRNA) sequences were designed using the Gene Art CRISPR gRNA Design Tool (Thermo Fisher Scientific) and synthesized using the Gene Art Precision gRNA Synthesis Kit (Thermo Fisher Scientific). The gRNAs (300 ng) were incubated with 1.5 μg of Gene Art Platinum Cas9 nuclease (Thermo Fisher Scientific) and then transfected into MKN1 cells stably expressing luciferase. Genomic cleavage efficiency was measured 72 h after transfection using the Gene Art Genomic Cleavage Detection Kit (Thermo Fisher Scientific) by the fragmentation patterns of bands in agarose gels. Single-cell clones were obtained by limiting dilution. In all cell lines, successful *NPTXR* KD, KO, or overexpression was confirmed by western blot analysis.

*Proliferation, Apoptosis, and Cell Cycle Assays*

Cell proliferation was analyzed using the Cell Counting Kit-8 (CCK-8) assay (Dojindo Molecular Technologies, Inc., Kumamoto, Japan). Cells (5 × 10^3^/well) were incubated in serum-free Dulbecco’s modified Eagle’s medium (Sigma-Aldrich, St. Louis, MO, USA) and proliferation was measured on days 1, 3, and 5. Apoptosis was measured using an annexin V-Alexa Fluor 568 conjugate (A13202, Thermo Fisher Scientific) according to the manufacturer’s protocol. Parental, *NPTXR*-KO, or ultraviolet-light irradiated (120 s; positive control) MKN1 cells (10^5^ in 100 μL) were placed on cover slips, mixed with 10 μL of annexin V conjugate, and incubated at room temperature for 15 min. Cells were visualized using an FSX100 (Olympus, Tokyo, Japan) fluorescence microscope. The number of annexin V-positive cells was determined from eight randomly selected fields.

Total caspase activity was measured using a Muse Multi-caspase kit (MCH100109, Merck Millipore, Billerica, MA, USA). Cells (10^5^ /mL) were incubated with 5 μL of Muse Multi-Caspase reagent and 150 μL of Muse Caspase 7-amino-actinomycin D (7-AAD) for 30 min at 37°C and then analyzed using the Muse Cell Analyzer (Merck Millipore). A Caspase Colorimetric Assay Kit (BioVision, Milpitas, CA, USA) was used to measure the activity of caspase-3, 8, 9, and -12 individually. Cells (5 × 10^4^) were lysed, incubated with a p-nitroaniline-conjugated, caspase-specific peptide, and the absorbance at 450 nm was measured using a spectrophotometer.

The Muse MitoPotential kit (Merck Millipore) was used to assess the mitochondrial membrane potential. Cells (10^5^) were incubated with Muse MitoPotential solution for 20 min at 37°C, treated with 5 μL of 7-AAD (Merck Millipore), and analyzed using a Muse Cell Analyzer (Merck Millipore). Cell cycle progression was analyzed using a Muse Cell Cycle Kit (Merck Millipore). Cells (10^6^) were incubated, fixed in 70% ethanol at −20°C for 5 h, mixed with 200 μL of Muse cell cycle reagent, and incubated for 30 min in the dark at room temperature. The number of cells in G0/G1, S, and G2/M phases of the cell cycle was determined using the Muse Cell Analyzer (Merck Millipore). To verify the results of the Muse assay, we also analyzed the cell cycle using a Cell Cycle Assay Cell-Clock Kit (Biocolor, Carrickfergus, UK) according to the manufacturer’s protocol. The cells were visualized using bright-field microscopy and quantified with ImageJ v.1.60.

*Cell Migration, Invasion, and Adhesion Assays*

Cell migration was assessed using wound-healing assays. In brief, cells were seeded into culture inserts of a 60-mm diameter dish (ibidi GmbH, Martinsried, Germany) to establish wound gaps of a defined width and incubated for 16 h. The insert was removed, and the wound width was measured at 100-mm intervals (20 measurements per well, 40× magnification). Cell invasion was measured using BioCoat Matrigel invasion chambers (BD Biosciences, Bedford, MA, USA) according to the manufacturer’s protocol. Invading cells in eight randomly selected fields were counted using a light microscope (200× magnification). The CytoSelect 48-Well Cell Adhesion Assay (Cell Biolabs, San Diego, CA, USA) was used to determine cell adhesion to the ECM proteins fibronectin, collagen I, collagen IV, laminin I, and fibrinogen. Cells were plated at 7.5 × 10^4^/well and incubated for 1 h before absorbance at 560 nm was measured.

*Aldehyde Dehydrogenase (ALDH) Assay*

ALDH was measured using the ALDEFLUOR fluorescent reagent system (Stem Cell Technologies, Vancouver, Canada), according to the manufacturer’s instructions. Briefly, MKN1 cells (10^5^/mL) were resuspended in kit buffer, mixed with the ALDH inhibitor N,N-diethylaminobenzaldehyde (25 μM), and then incubated with the activated ALDEFLUOR substrate boron-dipyrromethene aminoacetaldehyde (1.5 μM) for 45 min at 37°C. The cells were then washed and the proportion of ALDH-positive cells were determined using a FACSCalibur flow cytometer (BD Biosciences). Data were analyzed using CellQuestPro software (BD Biosciences).

*5-Fluorouracil (5-FU) and Cisplatin Sensitivity Assay*

MKN1 cells were incubated (5 ×10^3^/well) for 72 h with phosphate buffered saline (PBS) (control), 0.01, 0.1, 1, 10, and 100 μ/mL of 5-FU or 0.008, 0.04, 0.02, 1, 5, 25, and 125 μg/mL of cisplatin. Cell viability was measured using the CCK-8 assay, and the data are expressed as the ratio of absorbance at 450 nm of the test sample divided by the absorbance of the control sample.

*Mouse Subcutaneous Xenograft Model*

The Animal Research Committee of Nagoya University approved all animal experiments (approval number 31370). Cells (10^6^/injection) were resuspended in 100 µL of a 1:1 mixture of PBS and Matrigel (BD Biosciences) and injected subcutaneously (s.c.) into both flanks of 9-week-old male BALB/c nu/nu mice (n = 3/group; Japan SLC, Inc. Hamamatsu, Japan). Tumor growth was measured weekly, and the mice were euthanized at 8 weeks after injection. Tumor volume (TV) was calculated as: TV (mm^3^) = d2 × D/2, where d and D are the shortest and longest diameters, respectively. Measurements were taken by an investigator blinded to the experimental conditions.

*Generation of Rabbit Anti-NPTXR pAbs*

Two anti-NPTXR pAbs (pAb-1 and pAb-2) were generated by immunizing rabbits with synthetic peptides containing NPTXR epitopes predicted to be immunogenic by *in silico* analysis. Peptides were synthesized by solid-phase peptide synthesis with the fluorenyl-methoxy-carbonyl method (CESGLPRGLQGAGPRRDT for pAb-1 and KERVALSHSSRRQRQEVE for pAb-2).

*Immunofluorescence Microscopy*

Cells were seeded on coverslips and fixed with 4% paraformaldehyde for 20 min at room temperature, washed three times with PBS, blocked in PBS containing 1% BSA and 0.05% Tween-20 for 20 min, and then incubated with anti-NPTXR pAb-1 overnight. The cells were washed three times with PBS and then incubated with Alexa Fluor 488-conjugated anti-rabbit IgG (H+L) secondary Ab (Cell Signaling Technology, Danvers, MA, USA) for 1 h at room temperature. Nuclei were stained with 4',6‑diamidine‑2'‑phenylindole dihydrochloride (DAPI; Invitrogen, Carlsbad, CA, USA). Cell were visualized and quantified using a BZ-X800 fluorescence microscope system (Keyence, Itasca, IL, USA).

*Immunohistochemistry (IHC)*

NPTXR protein expression was analyzed in 80 formalin-fixed and paraffin-embedded sections of well-preserved tissues from patients with GC. Anti-NPTXR pAb-1 was diluted 1:100 in Ab diluent (Dako, Glostrup, Denmark) and incubated with the sections for 1 h at the room temperature. After washing, the secondary Ab was added and antigen-antibody complexes were visualized using liquid 3,30-diaminobenzidine (Nichirei, Tokyo, Japan) after a 1 min incubation. Specimens were analyzed by two independent observers who were blinded to the status of the samples.

*Generation of mAbs Against NPTXR*

Three 6-week-old female BALB/c mice were immunized s.c. twice at 3-week intervals with 40 μg of peptide CESGLPRGLQGAGPRRDT and then once more 3 weeks later with 40 μg peptide delivered by intraperitoneal (i.p.) injection. Two weeks later, the mice were bled and serum Ab titers were measured using an enzyme-linked immunosorbent assay (ELISA) with the immunizing peptide as the coating antigen. Sera prepared on day 0 (preimmunization) served as the negative control. The mouse with the highest Ab titer was then boosted again i.p. with 40 mg peptide, and the mouse was euthanized 1 week later. Splenocytes were prepared, fused with P3U1 myeloma cells, and the cell suspension was dispensed into 96-well plates and incubated at 37°C for 3 days. Supernatant samples were screened for anti-NPTXR Abs using the ELISA described above, and hybridoma cells from positive wells were cloned by limiting dilution in aminopterin-free selection medium. Ab-containing supernatants were also tested for their ability to inhibit GC cell proliferation. The best three clones based on Ab titer and inhibitory activity were selected for further evaluation and designated anti-NPTXR mAb-1, -2, and -3. The mAbs were stored at −20°C.

*Characterization of Anti-NPTXR mAbs*

NPTXR mAb-1 wase further characterized by epitope mapping and Ab-dependent cell-mediated cytotoxicity (ADCC) assay. Epitope mapping was performed using a competition ELISA in which 26 15-mer peptides encompassing the 40-amino acid sequence IRELTGKLGRCESGLPRGLQGAGPRRDTMADGPWDSPALI (position 151–190 of NPTXR) were coated in ELISA plates (100 ng/well) and incubated overnight at 4°C. The wells were then blocked in PBS containing 0.05% Tween 20 and 3% BSA at 37°C for 1 h, washed, and incubated at 37°C for 1 h. After washing, color was developed by addition of 3,3′,5,5′-tetramethylbenzidine substrate for 10 min, the reaction was stopped by addition of 3.5N NaOH, and the absorbance at 405 nm was measured. Absorbance values were compared to identify the peptide sequence bound with the highest avidity by the mAb.

Functional activity of the mAb-1 was determined using an ADCC Reporter Bioassay Kit (Promega, Madison, WI, USA) according to the manufacturer’s instructions. In brief, 10^4^ MKN1 cells were dispensed into 96-well plates with 25 µL ADCC assay buffer plus 25 µL of mAb-1 or control anti-CD20 antibody. Reporter cells (7.5 × 10^4^/well, Jurkat V variant) were added to the wells and incubated for 6 h at 37°C. Luciferase Assay Reagent (Promega) was added at 75 µL/well and the signal at 450 nm was measured.

*Mouse Xenograft Model of GC Peritoneal Metastasis*

MKN1 cells stably expressing luciferase (10^6^ cells/mouse) were injected into peritoneal cavity (i.p.) of 10-week-old male BALB/c nu/nu mice (n = 4) to establish a model of GC metastasis to the peritoneum. The mice were then injected i.p. twice a week for 6 weeks with 6 μg of normal mouse IgG (control; 140-09511, FUJIFILM Wako Pure Chemical Corporation, Tokyo, Japan), or anti-NPTXR pAb-1, pAb-2, mAb-1, mAb-2, or mAb-3. The mice were monitored for clinical signs, skin appearance, food intake, and body weights to assess general health. Growth of GC tumors in the peritoneal cavity was assessed using non-invasive imaging with In Vivo Imaging System (IVIS) Lumina (Xenogen, Alameda, CA, USA) and Living Image Ver.2.6 software (Xenogen). At 3 weeks after cell injection, the mice were injected i.p. with d-luciferin (150 mg/kg; Summit Pharmaceuticals International Corporation, Tokyo, Japan), and luciferase activity was measured using the IVIS 15 min later. Mice were euthanized 3 weeks after imaging (6 weeks after cell injection), the peritoneal cavities were examined, and peritoneal nodules were collected.

*Profiling of Intracellular Signaling*

Intracellular signaling in GC cells was evaluated using several methods. Phosphorylation of 1006 unique sites in 409 signaling proteins (including AKT, MAPK, NF-κB, and JAK–STAT signaling pathways) using the PTMScan® Direct Multi-Pathway Kit (Cell Signaling Technology), which combines antibody enrichment of post-translationally modified peptides with liquid chromatography–mass spectrometry-based detection and quantification.

Pull-down assay was performed to assess activation of small GTPases using an Arf6 Activation Assay Kit and Pan-Ras Activation Assay Kit (STA-407-6 and STA-400, respectively; Cell Biolabs, San Diego, CA, USA) according to the manufacturer’s protocols. In brief, cell lysates were incubated with agarose beads coupled to the protein-binding domain of GGA3 to selectively pull down the active forms of the small GTPases. The precipitated GTP-Arf/Pan-Ras proteins were detected by western blot analysis.

A Muse MAPK Activation Dual Detection Kit and a Muse PI3K Activation Dual Detection Kit (Merck Millipore) were used according to the manufacturer’s instructions to measure levels of phosphorylated ERK and AKT in GC cells. Data were analyzed using the Muse Cell Analyzer (Merck Millipore).

*Generation of Nptxr^−/−^ Mice*

*Nptxr^−/−^* mice were generated using the CRISPR/Cas9 system.[[4](#_ENREF_4)] In brief, Cas9 mRNA and sgRNAs were microinjected into fertilized embryos of C57BL/6J mice to induce nonhomologous end joining. Mutations in the *Nptxr* allele were confirmed by direct sequencing (Eurofins Genomics Co Ltd, Tokyo, Japan). Mice were genotyped 4 weeks after birth using PCR (primers: *Nptxr* forward, 5′-ATCCAGTCACCTCCTTCTGAGTG-3′ and *Nptxr* reverse, 5′-CTCTGCAGCAGCAACAGCTC-3′). The product sizes for the targeted and wildtype alleles were approximately 500 and 700 base pairs, respectively. PCR was performed using the GeneAmp PCR System 9700 (Applied Biosystems). *Nptxr^−/−^* mice were generated by crossing *Nptxr^+/−^* mice, and wildtype littermates were used as the controls for experiments. Mice were maintained in specific pathogen-free conditions at the Laboratory Animal Science of Nagoya University Graduate School of Medicine. Appearance and body weight were monitored for 8 weeks, and the development of major organs (macroscopic appearance and histology) and blood tests (blood counts and biochemistry) were evaluated at 8 weeks after birth in groups of *Nptxr*^+/+^, *Nptxr*^+/−^, and *Nptxr^−/−^*mice (n = 8 each). Because *NPTXR* is abundantly expressed in the nervous system,[[5](#_ENREF_5)] we also assessed general motor coordination and motor ability using the rotarod test (Economex Rotarod; Columbus Instruments, Columbus, OH, USA) when the mice were 6–8 weeks of age.[[6](#_ENREF_6)] Three tests were performed (n = 10 mice/group) to measure the time spent on the rod, and the longest time was recorded. An arbitrary time limit of 300 s was set for the experiment.

*Statistical Analysis*

The significance of the difference between two variables was assessed using Spearman’s rank correlation coefficient. The Mann–Whitney test was used to compare the differences between two groups. Overall survival (OS) and disease-free survival (DFS) rates were calculated using the Kaplan–Meier method, and the difference between groups was analyzed using the log-rank test. Goodness-of-fit was assessed by calculating the area under the curve (AUC) of the receiver operating characteristic (ROC) curve, and the optimal cut-off value was determined using the Youden index. Associations between *NPTXR* mRNA levels and clinical variables were evaluated using the chi-square test. The univariate Cox proportional hazards model was used to evaluate the hazard ratio for OS relative to each variable. Variables with P < 0.05 were included in the multivariate analysis to identify independent predictive factors. Statistical analyses were performed using JMP 13 software (SAS Institute Inc., Cary, NC). P < 0.05 was considered statistically significant.

**References**

1. Szasz AM, Lanczky A, Nagy A et al. Cross-validation of survival associated biomarkers in gastric cancer using transcriptomic data of 1,065 patients. Oncotarget 2016; 7: 49322-49333

2. Kanda M, Shimizu D, Tanaka H et al. Significance of SYT8 For the Detection, Prediction, and Treatment of Peritoneal Metastasis From Gastric Cancer. Annals of surgery 2018; 267: 495-503

3. Kanda M, Tanaka H, Shimizu D et al. SYT7 acts as a driver of hepatic metastasis formation of gastric cancer cells. Oncogene 2018, DOI: 10.1038/s41388-018-0335-8

4. Wang H, Yang H, Shivalila CS et al. One-step generation of mice carrying mutations in multiple genes by CRISPR/Cas-mediated genome engineering. Cell 2013; 153: 910-918

5. Yin GN, Lee HW, Cho JY et al. Neuronal pentraxin receptor in cerebrospinal fluid as a potential biomarker for neurodegenerative diseases. Brain research 2009; 1265: 158-170

6. Katsuno M, Adachi H, Kume A et al. Testosterone reduction prevents phenotypic expression in a transgenic mouse model of spinal and bulbar muscular atrophy. Neuron 2002; 35: 843-854
