# Supplementary material for: Therapeutic monoclonal antibody targeting of neuronal pentraxin receptor to control metastasis in gastric cancer
Source: Mol Cancer. 2020 Aug 26;19:131. doi: 10.1186/s12943-020-01251-0 (PMC7448342; doi:10.1186/s12943-020-01251-0)
Supplement: Supplementary file 2 — Additional file 2: Table S1. Nucleotide sequences. [file 12943_2020_1251_MOESM2_ESM.doc]

|  | **Experiment** | **Sense** | **Sequence (5´ - 3´)** | **Product size** | **Annealing temperature** |
| --- | --- | --- | --- | --- | --- |
| ***NPTXR*** | qRT-PCR | forward | TCATTCTGGAGCTGGAGGAC | 95 bp | 60 °C |
|  |  | reverse | GGCAGCTGAGAGGTTCACA |  |  |
|  | gRNA | gRNA-1 target sequence | CGGTGCCGTCATCTGCATCA |  | |
|  |  | gRNA-2 target sequence | GTTCAGCCGCTTCCTGTGCA |  |  |
|  | Cleavage detection | Cleavage-1 forward | GCCTCACGCTGAAGTTCC | 294 bp | 64 °C |
|  |  | Cleavage-1 reverse | ACAGGAAGCGGCTGAACA |  |  |
|  |  | Cleavage-2 forward | CCTCACGCTGAAGTTCCTG | 426 bp | 66 °C |
|  |  | Cleavage-2 reverse | AGCTCACGGATGGTGTCCT |  |  |
|  | siRNA | si*NPTXR* -1 | CCAUGGAGCUGCUGAUCAA |  | |
|  |  | si*NPTXR* -2 | GGCAGGAAGUGGAAAAGGA |  |  |
| ***GAPDH*** | qRT-PCR | forward | GAAGGTGAAGGTCGGAGTC | 226 bp | 60 °C |
|  |  | probe | CAAGCTTCCCGTTCTCAGCC |  |  |
|  |  | reverse | GAAGATGGTGATGGGATTTC |  |  |

**Supplemental Table 1.** Nucleotide sequences

*NPTXR*, neuronal pentraxin receptor; *GAPDH*, glyceraldehyde-3-phosphate dehydrogenase; *qRT-PCR*, quantitative real-time reverse-transcription polymerase chain reaction; *gRNA*, guide RNA; siRNA, small interfering RNA.
