# Supplementary material for: Therapeutic monoclonal antibody targeting of neuronal pentraxin receptor to control metastasis in gastric cancer
Source: Mol Cancer. 2020 Aug 26;19:131. doi: 10.1186/s12943-020-01251-0 (PMC7448342; doi:10.1186/s12943-020-01251-0)
Supplement: Supplementary file 3 — Additional file 3: Figure S1. Extraction diagram of candidate genes identified by global expression analysis. Sixteen patients were categorized into four groups (4 patients for each) according to clinical courses as follows: group 1, no recurrences for > 5 years; group 2, hepatic-confined metastasis within 2 years after surgery; group 3, peritoneal metastasis within 2 years after surgery; and group 4, nodal metastasis within 2 years after surgery. Global expression profiling was conducted to compare the expression levels of 57,749 genes between each cluster. Among them, 14 we considered 14 genes as candidates shown in Supplemental Table 2. [file 12943_2020_1251_MOESM3_ESM.pdf]

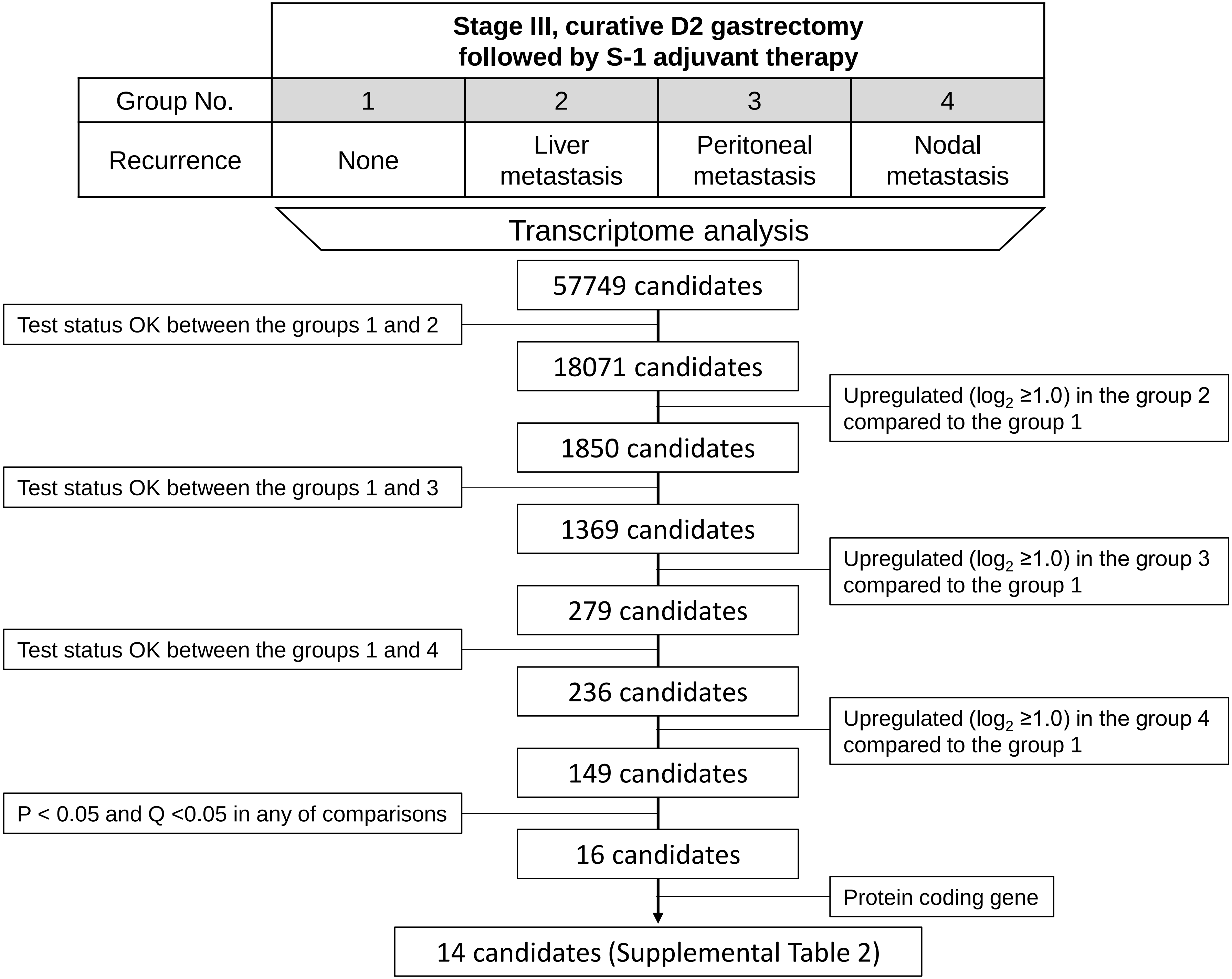

Stage III, curative D2 gastrectomy followed by S-1 adjuvant therapy
Group No.
1
2
3
4
Recurrence
None
Liver metastasis
Peritoneal metastasis
Nodal metastasis
Transcriptome analysis
57749 candidates
Test status OK between the groups 1 and 2
18071 candidates
Upregulated (log_2 ≥1.0) in the group 2 compared to the group 1
1850 candidates
Test status OK between the groups 1 and 3
1369 candidates
Upregulated (log_2 ≥1.0) in the group 3 compared to the group 1
279 candidates
Test status OK between the groups 1 and 4
236 candidates
Upregulated (log_2 ≥1.0) in the group 4 compared to the group 1
149 candidates
P < 0.05 and Q <0.05 in any of comparisons
16 candidates
Protein coding gene
14 candidates (Supplemental Table 2)
