# Supplementary material for: Therapeutic monoclonal antibody targeting of neuronal pentraxin receptor to control metastasis in gastric cancer
Source: Mol Cancer. 2020 Aug 26;19:131. doi: 10.1186/s12943-020-01251-0 (PMC7448342; doi:10.1186/s12943-020-01251-0)
Supplement: Supplementary file 4 — Additional file 4: Table S2. List of candidate genes upregulated in gastric cancer tissues of patients with metachronous metastasis. [file 12943_2020_1251_MOESM4_ESM.docx]

**Supplemental Table 2.** List of candidate genes upregulated in gastric cancer tissues of patients with metachronous metastasis

| **Biological function** | **Symbol** | **Full name** | **Location** | **H-meta/**  **No meta** | **P-rec/ No meta** | **N-rec/ No meta** |
| --- | --- | --- | --- | --- | --- | --- |
| Neuronal transporter | *NPTXR* | neuronal pentraxin receptor | 22q13.1 | 4.388 | 2.265 | 1.71 |
| Cell adhesion protein | *MSLN* | mesothelin | 16p13.3 | 1.318 | 2.423 | 1.458 |
| Cytokine receptor | *IL22RA1* | interleukin 22 receptor subunit alpha 1 | 1p36.11 | 1.586 | 1.276 | 2.706 |
| Metabolic enzyme | *CYP2B6* | cytochrome P450 family 2 subfamily B member 6 | 19q13.2 | 4.128 | 1.953 | 3.411 |
|  | *CYP2W1* | cytochrome P450 family 2 subfamily W member 1 | 7p22.3 | 7.432 | 1.468 | 1.809 |
|  | *DPEP1* | dipeptidase 1 | 16q24.3 | 6.219 | 1.652 | 5.527 |
|  | *XPNPEP2* | X-prolyl aminopeptidase 2 | Xq26.1 | 2.676 | 1.285 | 3.441 |
| Neuronal acetylcholine and nicotine receptor | *CHRNB2* | cholinergic receptor nicotinic beta 2 subunit | 1q21.3 | 2.45 | 4.879 | 5.361 |
| Regulator of osteoclast differentiation | *TMEM178B* | transmembrane protein 178B | 7q34 | 1.228 | 1.887 | 3.812 |
| Regulator of neurotransmitter | *SYP* | synaptophysin | Xp11.23 | 1.289 | 2.07 | 3.269 |
|  | *BSN* | bassoon presynaptic cytomatrix protein | 3p21.31 | 2.183 | 3.316 | 4.097 |
| Secreted lipid-binding protein | *APOC2* | apolipoprotein C2 | 19q13.32 | 4.015 | 1.859 | 1.152 |
| Transcription factor | *PROX1* | prospero homeobox 1 | 1q32.3 | 2.534 | 2.109 | 3.03 |
|  | *ASCL2* | achaete-scute family bHLH transcription factor 2 | 11p15.5 | 3.967 | 1.92 | 5.055 |

H-meta, hepatic metastasis; No meta, no metastasis; P-meta. Peritoneal metastasis; L-meta, lymph node metastasis.
