# Supplementary figures and images for: Therapeutic monoclonal antibody targeting of neuronal pentraxin receptor to control metastasis in gastric cancer
Source: Mol Cancer. 2020 Aug 26;19:131. doi: 10.1186/s12943-020-01251-0 (PMC7448342; doi:10.1186/s12943-020-01251-0)

**A**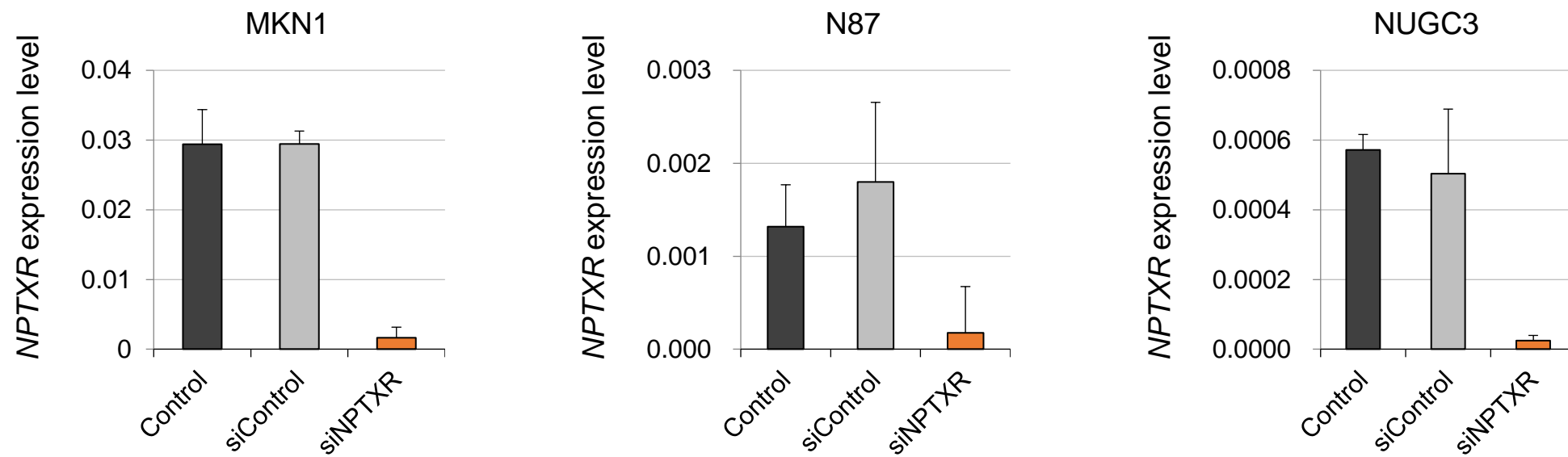**B**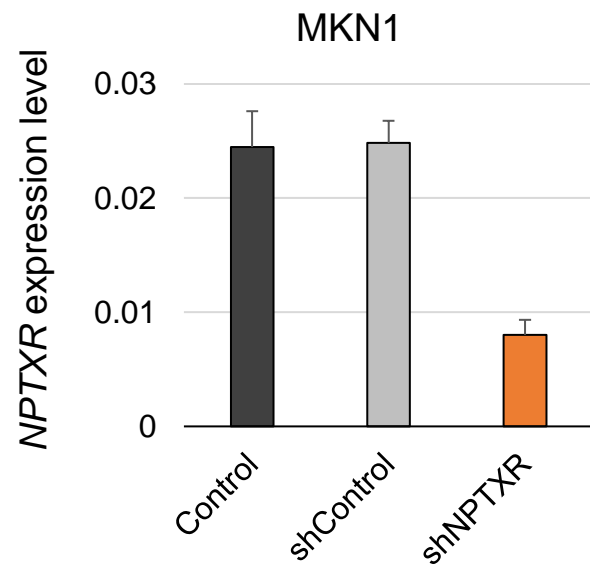**C**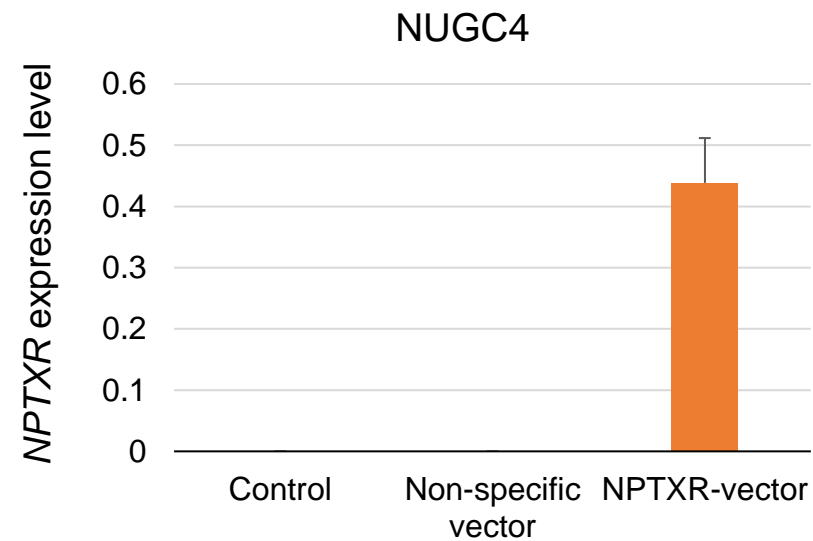

Supplement: Supplementary file 5 — Additional file 5: Figure S2. Verification of NPTXR knockdown or overexpression in GC cells. a-c qRT-PCR analysis of NPTXR mRNA expression in MKN1, N87, and/or NUGC3 cells with siRNA-mediated knockdown (a), shRNA-mediated knockdown (b), or overexpression (c) of NPTXR. *P < 0.05. Mean ± standard deviation. [file 12943_2020_1251_MOESM5_ESM.pdf]

**A**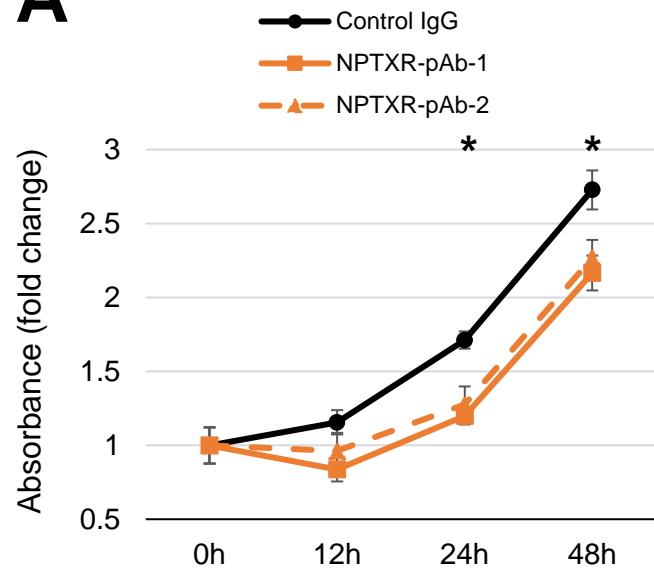**B**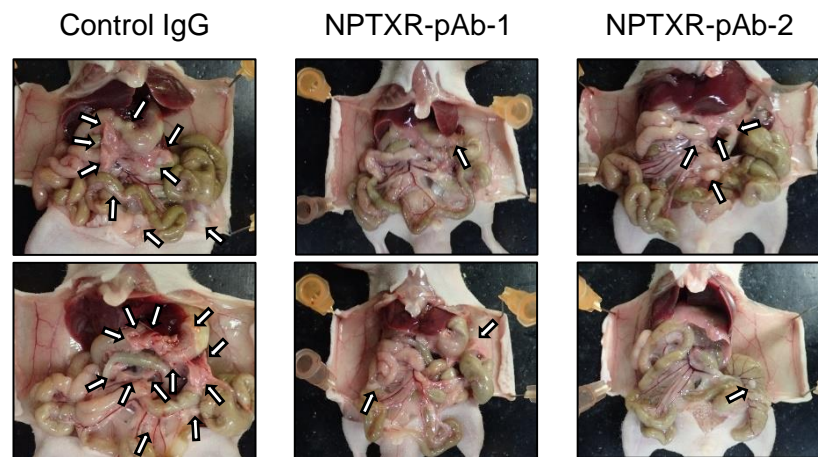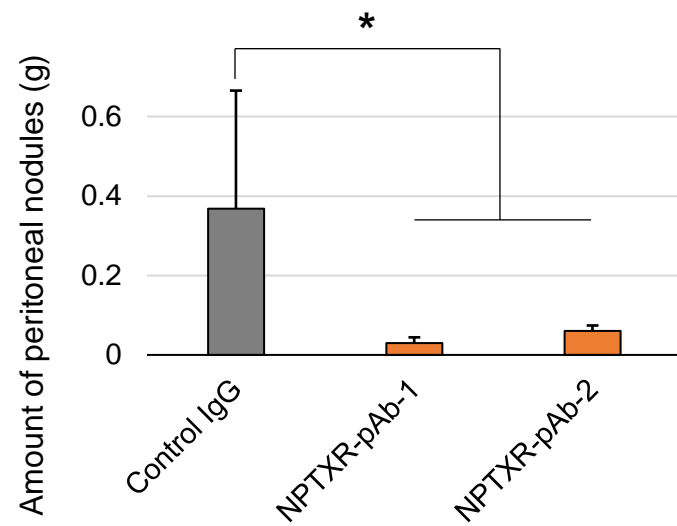**C**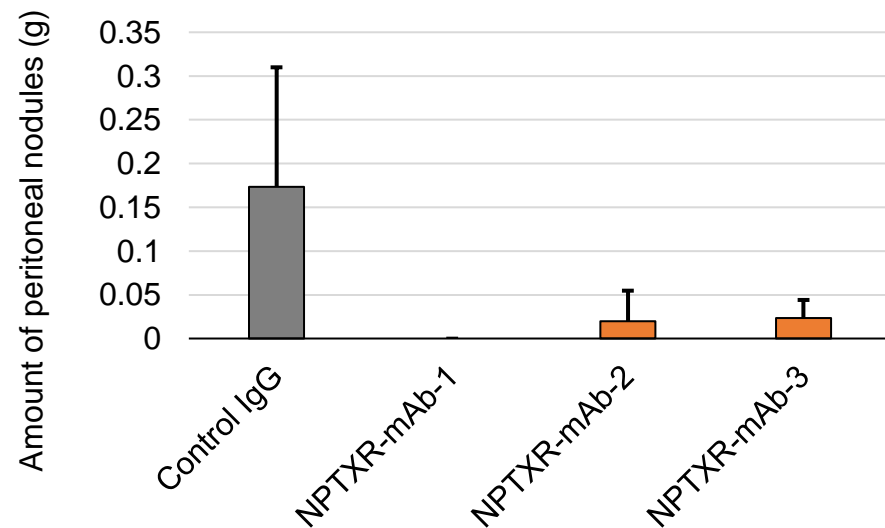

Supplement: Supplementary file 6 — Additional file 6: Figure S3. Characterization of polyclonal anti-NPTXR Abs effects in vitro and in vivo. a CCK-8 proliferation assay of MKN1 cells incubated with pAbs in vitro. b Therapeutic effects of intraperitoneal administration of anti-NPTXR polyclonal antibodies. Macroscopic appearance of peritoneal nodules 6 weeks after injection of MKN1 cells and treatment with control IgG or NPTXR-pAbs. c Total volumes of peritoneal nodules collected from BALB/c nu/nu mice 6 weeks after injection of MKN1 cells and treatment with control IgG or NPTXR-mAb-1, − 2, or − 3. *P < 0.05. Mean ± standard deviation. [file 12943_2020_1251_MOESM6_ESM.pdf]

**A**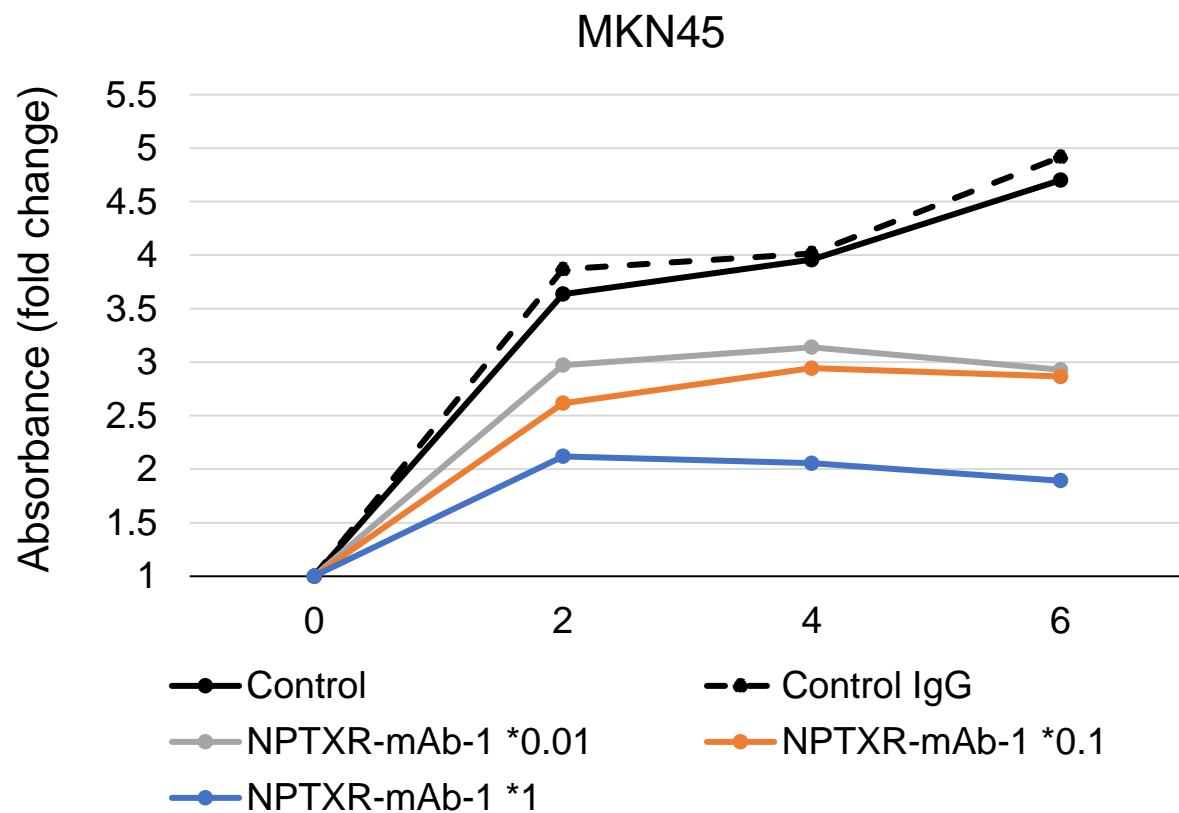**B**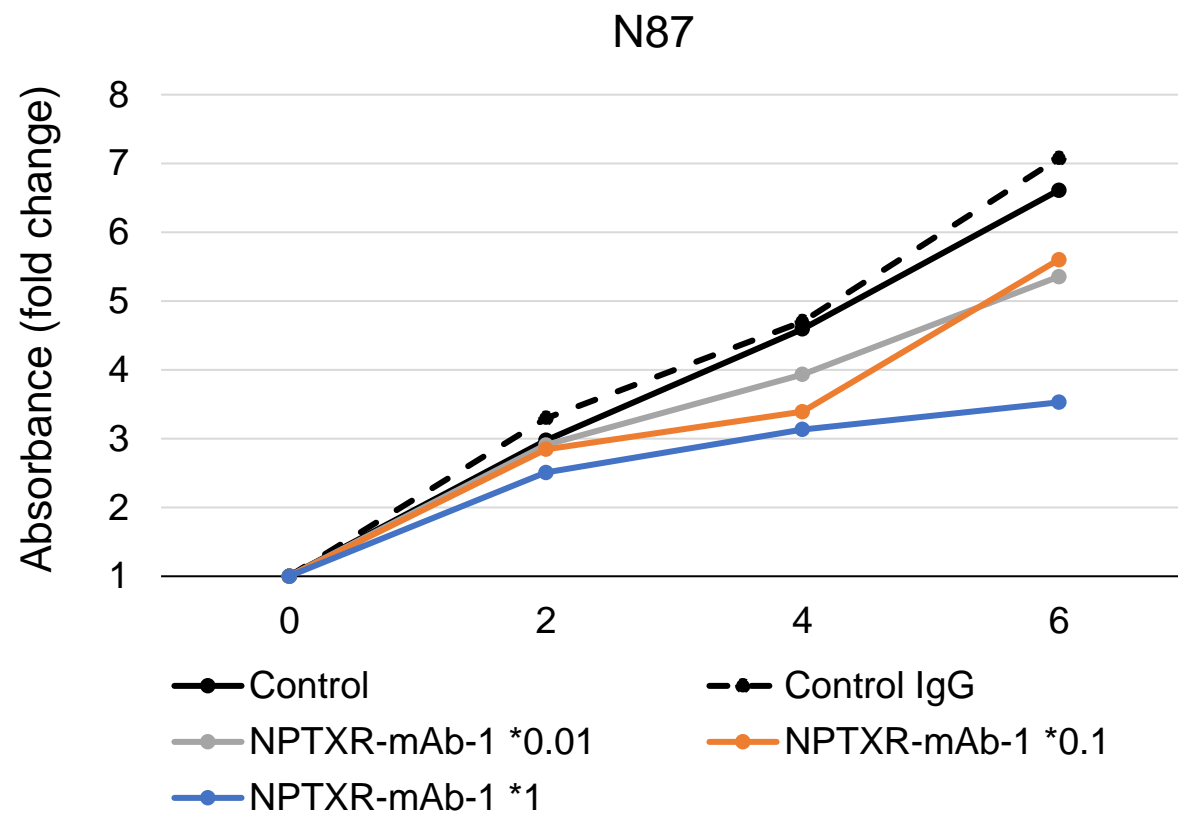

Supplement: Supplementary file 7 — Additional file 7: Figure S4. Effects of NPTXR-mAb-1 on gastric cancer cells. CCK-8 proliferation assay of MKN45 (a) and N87 (b) cells incubated with NPTXR-mAb-1 in dose-dependent manner. [file 12943_2020_1251_MOESM7_ESM.pdf]

**A**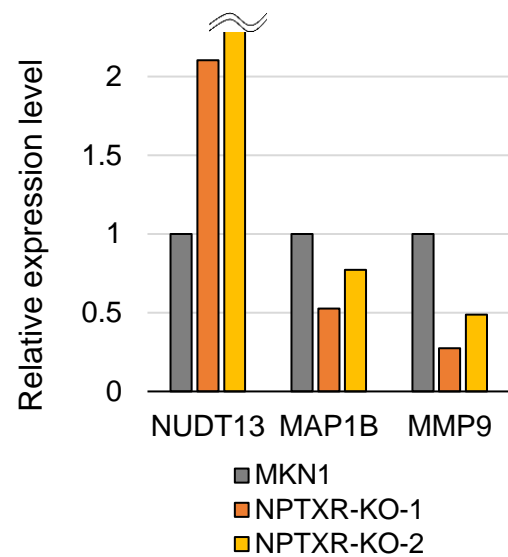**B**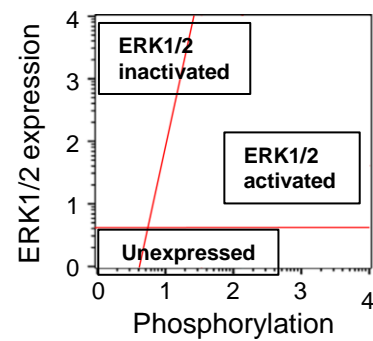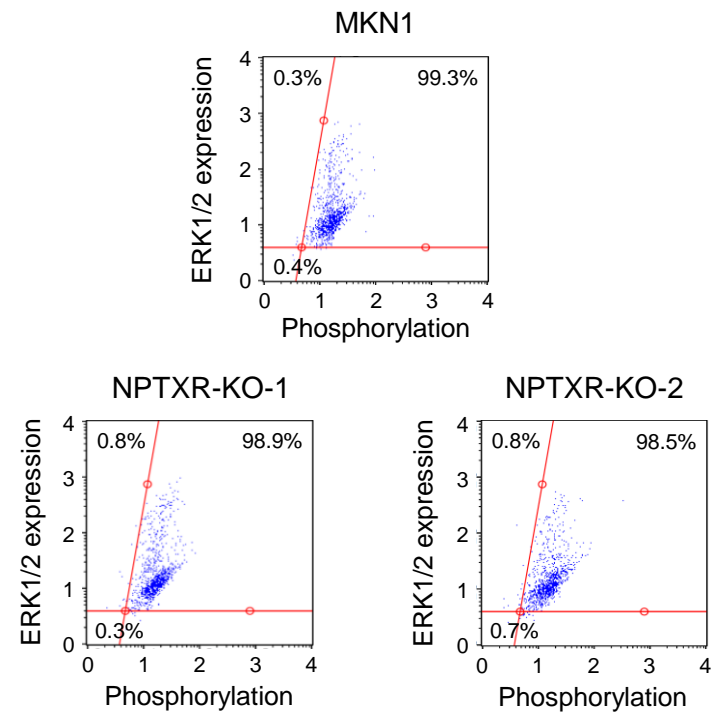**C**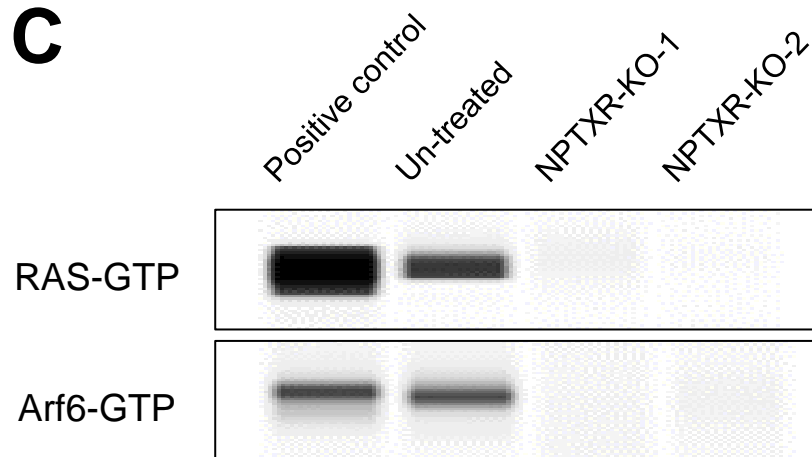

Supplement: Supplementary file 8 — Additional file 8: Figure S5. a qRT-PCR analysis of NUDT13, MAP 1B, MMP9 mRNA levels in parental MKN1, NPTXR-KO-1 and -KO-2 cells. b Flow cytometric analysis of the frequency parental and NPTXR-KO MKN1 cells with phosphorylated ERK1/2. c Pull-down of small GTPases (Ras and Arf6) in parental and NPTXR-KO MKN1 cells. [file 12943_2020_1251_MOESM8_ESM.pdf]

**A**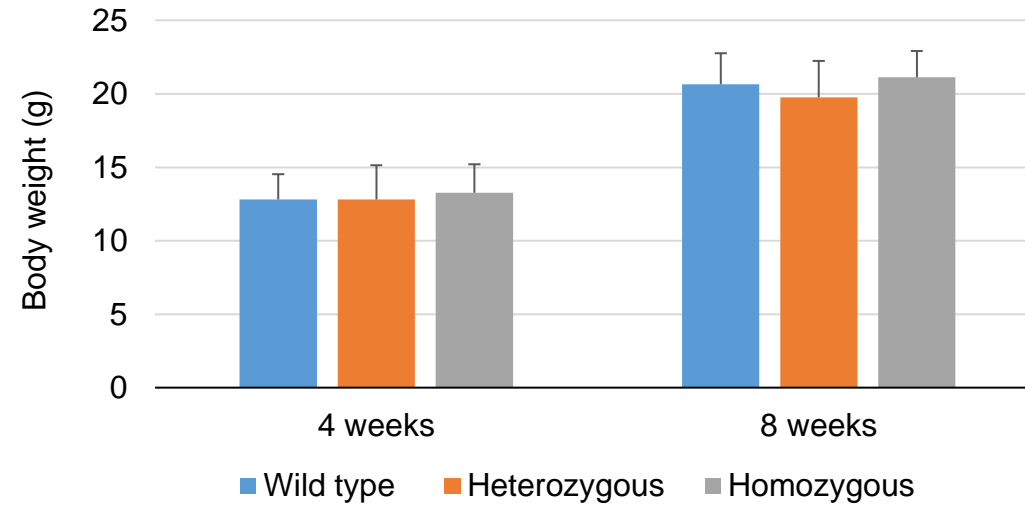**B**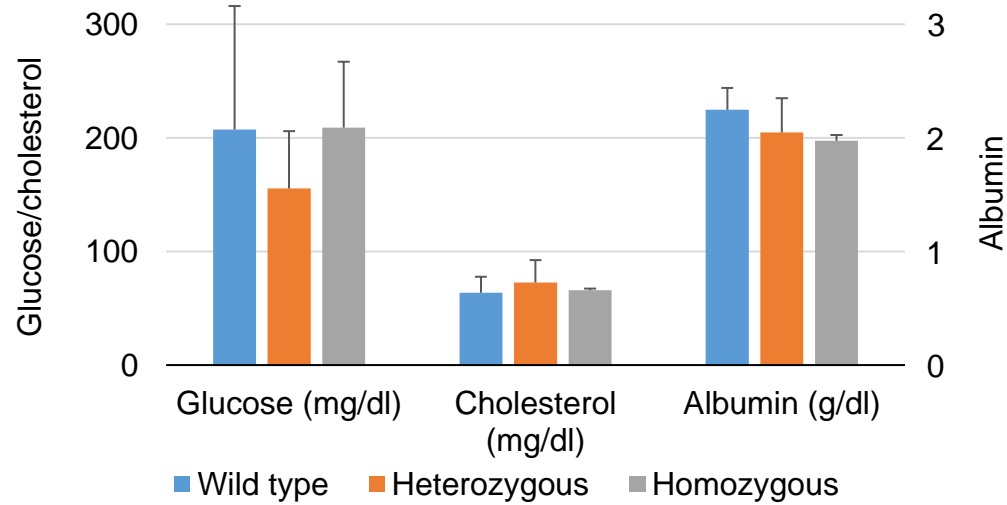**C**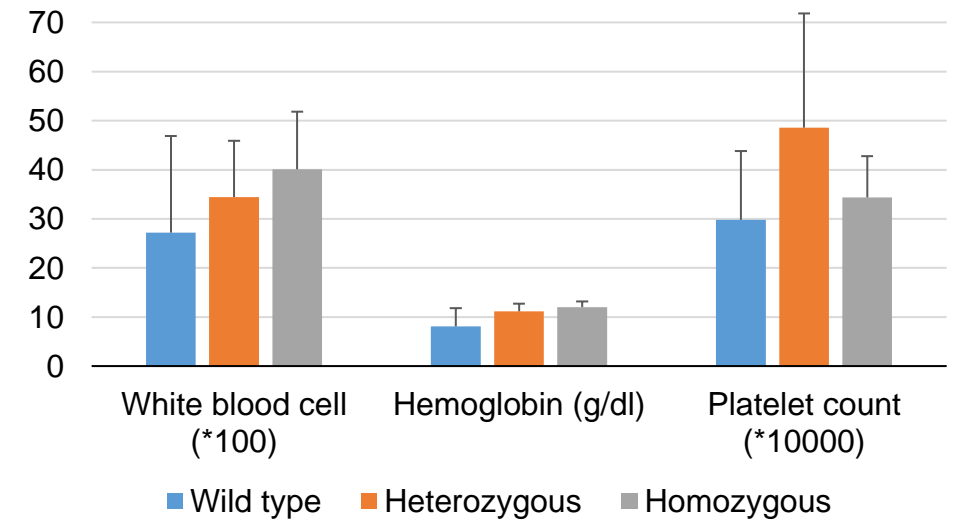

Supplement: Supplementary file 9 — Additional file 9: Figure S6. a Body weights of Nptxr+/+, Nptxr+/−, and Nptxr−/− mice at 4 and 8 weeks after birth. b and c Metabolic (b) and hematological (c) tests in Nptxr+/+, Nptxr+/−, and Nptxr−/− mice. [file 12943_2020_1251_MOESM9_ESM.pdf]
