# Supplementary material for: Therapeutic monoclonal antibody targeting of neuronal pentraxin receptor to control metastasis in gastric cancer
Source: Mol Cancer. 2020 Aug 26;19:131. doi: 10.1186/s12943-020-01251-0 (PMC7448342; doi:10.1186/s12943-020-01251-0)
Supplement: Supplementary file 10 — Additional file 10: Figure S7. a ROC curve analysis of the ability of NPTXR expression level in tissue specimens to predict peritoneal metastasis in GC patients. b Frequency of the site of initial recurrence in GC patients according to NPTXR expression level. c Disease-free survival rates in subgroups according to administration of adjuvant chemotherapy. [file 12943_2020_1251_MOESM10_ESM.pdf]

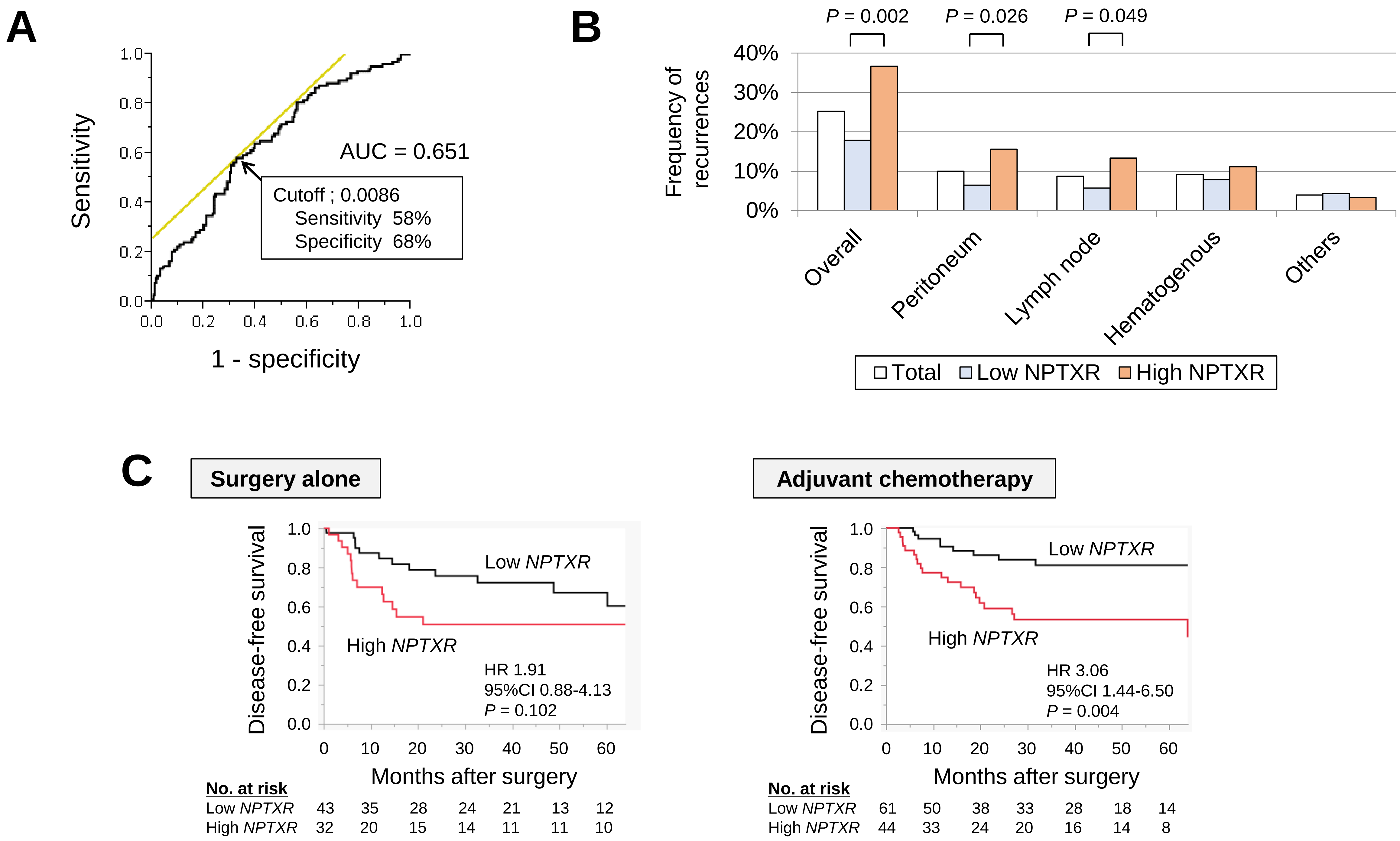
A
Sensitivity
1 - specificity
1.0
0.8
0.6
0.4
0.2
0.0
0.0
0.2
0.4
0.6
0.8
1.0
AUC = 0.651
Cutoff ; 0.0086
Sensitivity 58%
Specificity 68%
B
P = 0.002
P = 0.026
P = 0.049
Frequency of recurrences
40%
30%
20%
10%
0%
Overall
Peritoneum
Lymph node
Hematogenous
Others
Total
Low NPTXR
High NPTXR
C
Surgery alone
Disease-free survival
1.0
0.8
0.6
0.4
0.2
0.0
Low NPTXR
High NPTXR
HR 1.91
95%CI 0.88-4.13
P = 0.102
0
10
20
30
40
50
60
Months after surgery
No. at risk
Low NPTXR 43 35 28 24 21 13 12
High NPTXR 32 20 15 14 11 11 10
Adjuvant chemotherapy
Disease-free survival
1.0
0.8
0.6
0.4
0.2
0.0
Low NPTXR
High NPTXR
HR 3.06
95%CI 1.44-6.50
P = 0.004
0
10
20
30
40
50
60
Months after surgery
No. at risk
Low NPTXR 61 50 38 33 28 18 14
High NPTXR 44 33 24 20 16 14 8
