# Supplementary material for: Therapeutic monoclonal antibody targeting of neuronal pentraxin receptor to control metastasis in gastric cancer
Source: Mol Cancer. 2020 Aug 26;19:131. doi: 10.1186/s12943-020-01251-0 (PMC7448342; doi:10.1186/s12943-020-01251-0)
Supplement: Supplementary file 11 — Additional file 11: Table S3. Patients’ clinical characteristics associated with NPTXR expression. [file 12943_2020_1251_MOESM11_ESM.docx]

**Supplemental Table 3.** Patients’ clinical characteristics associated with *NPTXR* expression

| **Variables** | **High *NPTXR***  **(n = 124)** | **Low *NPTXR***  **(n = 176)** | ***P*** |
| --- | --- | --- | --- |
| Age  < 70 year  ≥ 70 year | 70  54 | 103  73 | 0.721 |
| Sex  Male  Female | 80  44 | 136  40 | 0.016 |
| CEA (ng/ml)  ≤ 5  > 5 | 97  27 | 142  34 | 0.604 |
| CA19-9 (IU/ml)  ≤ 37  > 37 | 98  26 | 138  38 | 0.897 |
| Tumor location  Entire  Upper third  Middle third  Lower third | 14  26  35  49 | 8  47  61  60 | 0.073 |
| Tumor size (mm)  < 50  ≥ 50 | 51  73 | 84  92 | 0.257 |
| Macroscopic type  Borrmann type 4/5  Others | 24  100 | 17  159 | 0.017 |
| Tumor depth (UICC)  pT1-3  pT4 | 57  67 | 104  72 | 0.025 |
| Differentiation  Differentiated  Undifferentiated | 38  86 | 81  95 | 0.007 |
| Lymphatic involvement  Absent  Present | 15  109 | 26  150 | 0.504 |
| Vascular invasion  Absent  Present | 42  82 | 59  117 | 0.950 |
| Infiltrative growth type  Invasive growth  Expansive growth | 53  71 | 59  117 | 0.105 |
| Multifocal lesion  Absent  Present | 110  14 | 163  13 | 0.248 |
| Lymph node metastasis  Absent  Present | 32  92 | 60  116 | 0.123 |
| Peritoneal metastasis  Negative  Positive | 97  27 | 146  30 | 0.306 |
| Synchronous hepatic metastasis  Absent  Present | 118  6 | 170  6 | 0.537 |
| UICC stage  I  II  III  IV | 14  25  51  34 | 36  46  58  36 | 0.051 |

CEA, carcinoembryonic antigen; CA19-9, carbohydrate antigen 19-9; UICC, Union for International Cancer Control.
