# Supplementary material for: Therapeutic monoclonal antibody targeting of neuronal pentraxin receptor to control metastasis in gastric cancer
Source: Mol Cancer. 2020 Aug 26;19:131. doi: 10.1186/s12943-020-01251-0 (PMC7448342; doi:10.1186/s12943-020-01251-0)
Supplement: Supplementary file 12 — Additional file 12: Table S4. Prognostic factors of patients with resectable gastric cancer. [file 12943_2020_1251_MOESM12_ESM.doc]

**Supplemental Table 4. Prognostic factors of patients with resectable gastric cancer**

| Variables | | Univariate | | | Multivariable | | |
| --- | --- | --- | --- | --- | --- | --- | --- |
|  |  | Hazard ratio | 95% CI | *P* | Hazard ratio | 95% CI | *P* |
| Age | ≥ 70 years | 1.05 | 0.56 – 2.07 | 0.882 |  |  |  |
| Gender | Female | 1.10 | 0.54 – 2.12 | 0.774 |  |  |  |
| Tumor location | Lower third | 0.77 | 0.38 – 1.45 | 0.421 |  |  |  |
| Carcinoembryonic antigen | > 5 ng/ml | 1.39 | 0.60 – 2.86 | 0.420 |  |  |  |
| Carbohydrate antigen 19-9 | > 37 IU/ml | 2.47 | 1.17 – 4.84 | 0.019 | 1.43 | 0.66 – 2.88 | 0.347 |
| Tumor size | ≥ 50 mm | 3.06 | 1.60 – 6.23 | <0.001 | 2.14 | 1.11 – 4.39 | 0.023 |
| Macroscopic type | Borrmann 4/5 | 2.01 | 0.82 – 4.28 | 0.121 |  |  |  |
| Tumor depth | pT4, UICC | 3.65 | 1.95 – 7.07 | <0.001 | 1.80 | 0.90 – 3.74 | 0.099 |
| Tumor differentiation | Undifferentiated | 1.86 | 0.98 – 3.72 | 0.059 |  |  |  |
| Lymphatic involvement | Present | 5.40 | 1.65 – 33.3 | 0.003 | 1.81 | 0.24 – 9.08 | 0.525 |
| Vascular invasion | Present | 3.81 | 1.84 – 8.89 | <0.001 | 1.92 | 0.85 – 4.98 | 0.124 |
| Infiltrative growth type | Invasive | 2.21 | 1.17 – 4.11 | 0.015 | 1.27 | 0.63 – 2.56 | 0.503 |
| Multifocal lesion | Present | 1.33 | 0.48 – 5.51 | 0.621 |  |  |  |
| Lymph node metastasis | Present | 12.6 | 4.49 – 52.4 | <0.001 | 7.11 | 2.03 – 36.8 | <0.001 |
| *NPTXR* expression | High | 2.73 | 1.46 – 5.27 | 0.002 | 2.04 | 1.08 – 3.99 | 0.029 |

CI, confidence interval; UICC, Union for International Cancer Control.
